# Unexpected similarity between HIV-1 reverse transcriptase and tumor necrosis factor binding sites revealed by computer vision

**DOI:** 10.1186/s13321-021-00567-3

**Published:** 2021-11-23

**Authors:** Merveille Eguida, Didier Rognan

**Affiliations:** Laboratoire d’Innovation Thérapeutique, UMR 7200 CNRS, Université de Strasbourg, 67400 Illkirch, France

**Keywords:** Binding sites, Similarity, Point cloud registration

## Abstract

Rationalizing the identification of hidden similarities across the repertoire of druggable protein cavities remains a major hurdle to a true proteome-wide structure-based discovery of novel drug candidates. We recently described a new computational approach (ProCare), inspired by numerical image processing, to identify local similarities in fragment-based subpockets. During the validation of the method, we unexpectedly identified a possible similarity in the binding pockets of two unrelated targets, human tumor necrosis factor alpha (TNF-α) and HIV-1 reverse transcriptase (HIV-1 RT). Microscale thermophoresis experiments confirmed the ProCare prediction as two of the three tested and FDA-approved HIV-1 RT inhibitors indeed bind to soluble human TNF-α trimer. Interestingly, the herein disclosed similarity could be revealed neither by state-of-the-art binding sites comparison methods nor by ligand-based pairwise similarity searches, suggesting that the point cloud registration approach implemented in ProCare, is uniquely suited to identify local and unobvious similarities among totally unrelated targets.

**Supplementary Information:**

The online version contains supplementary material available at 10.1186/s13321-021-00567-3.

## Introduction

Among the many possible approaches to structure-based drug design [1, 2], inferring novel ligand properties from the large-scale comparison of their possible binding pockets gains popularity as the repertoire of protein cavities of known three-dimensional (3D) structures (pocketome) is constantly increasing, thereby offering unique opportunities to design ligands while simultaneously considering multiple targets [3]. The term ‘pocketome’ was first coined in 2004 by An et al. [4] to describe the universe of cavities located at the surface of macromolecules and capable of binding low molecular-weight ligands. A systematic survey of currently available three-dimensional structures [5], suggests that its size is estimated to ca. 250,000 pockets [6] out of which 10–15% are accommodating true drug-like compounds [7, 8]. Pocket locations can be inferred from the position of already-bound molecules or predicted on the fly, by one of the many available cavity detection methods [3, 9]. The pockeome space can then be searched by numerous computational tools [10] for similarity to any query cavity to predict evolutionary relationships and protein–ligand interactions [3]. The later application is notably of paramount importance to the drug discovery field as it may reveal hidden relationships for guiding the design of safer drug candidates with a precise control of selectivity [3] with respect to either on-targets (polypharmacology approach) [11] or off-targets (side effects mitigation) [12], in a time and cost-effective manner [13].

Currently available methods are generally able to detect global similarities between two druggable pockets from different proteins, and therefore permit to transfer drug-like compounds from one target space to another [3]. Identifying more subtle local similarities at the level of fragment-bound pockets remains a much more difficult problem [14] as it requires a searchable archive of fragment-bound subpockets [15–17] and a computational method focusing on local subpocket descriptors. Consequently, there are still very few reports of experimentally verified subpocket similarity examples that have enabled the transfer of chemical fragments across unrelated proteins [18]. To fill the need for local similarity searching methods while comparing pockets of different sizes, we developed a novel method (ProCare) [17] relying on point cloud registration, a numerical image processing to find a spatial transformation (e.g.*,* scaling, rotation and translation) that aligns two point clouds [19, 20]. ProCare uses as input a point cloud representation of the protein pocket or subpockets, where each point is annotated by eight possible pharmacophoric features (hydrophobic, aromatic, H-bond donor, H-bond acceptor, H-bond donor and acceptor, positive, negative, dummy) complementary to that of the pocket microenvironment [21]. Since ProCare uses local descriptors to compare and align binding subpockets, the method is particularly suited to fragment-based design strategies aimed at positioning fragments in subpockets of any druggable cavity.

While validating the method by focused benchmarking studies [17], we noticed some unexpected local similarity between subpockets from two unrelated proteins with 23% sequence identity: human tumor necrosis factor alpha (TNF-α) trimer [22] and human immunodeficiency virus type 1 reverse transcriptase (HIV-1 RT) [23]. On the one hand side, TNF-α is a homotrimeric pro-inflammatory cytokine involved in autoimmune disorders such as rheumatoid arthritis and Crohn's disease [24]. It is currently targeted by monoclonal antibodies preventing its recognition by TNF-α receptors (TNFR1 and TNFR2). To date, no small molecule TNF-α inhibitor has reached the market [22]. On the other side, HIV-1 RT is an enzyme used by the HIV virus to replicate its genome by first generating a complementary DNA from the viral RNA template. HIV-1 RT can be blocked by many marketed drugs [25] binding to either the catalytic site (nucleoside inhibitors, e.g. zidovudine) or a remote allosteric pocket (non-nucleoside inhibitors, e.g. efavirenz).

To exclude potential artifacts or biases and provide a strong statistical support to this initial prediction, we here systematically compared the inner cavity of three inhibitor-bound TNF-α trimer structures with 122 non-nucleoside inhibitor-bound HIV-1 RT X-ray structures. In a large majority of pairwise comparisons, the corresponding subpockets were deemed similar, a prediction that could be confirmed by biophysical experiments evidencing a direct micromolar binding of non-nucleoside HIV-1 RT inhibitors to human soluble TNF-α. Interestingly, this unexpected similarity could not be recovered by state-of-the-art cavity comparisons tools suggesting the unique ability of ProCare to delineate subtle local relationships between unrelated target cavities.

## Results and discussion

**Fig. 1:**
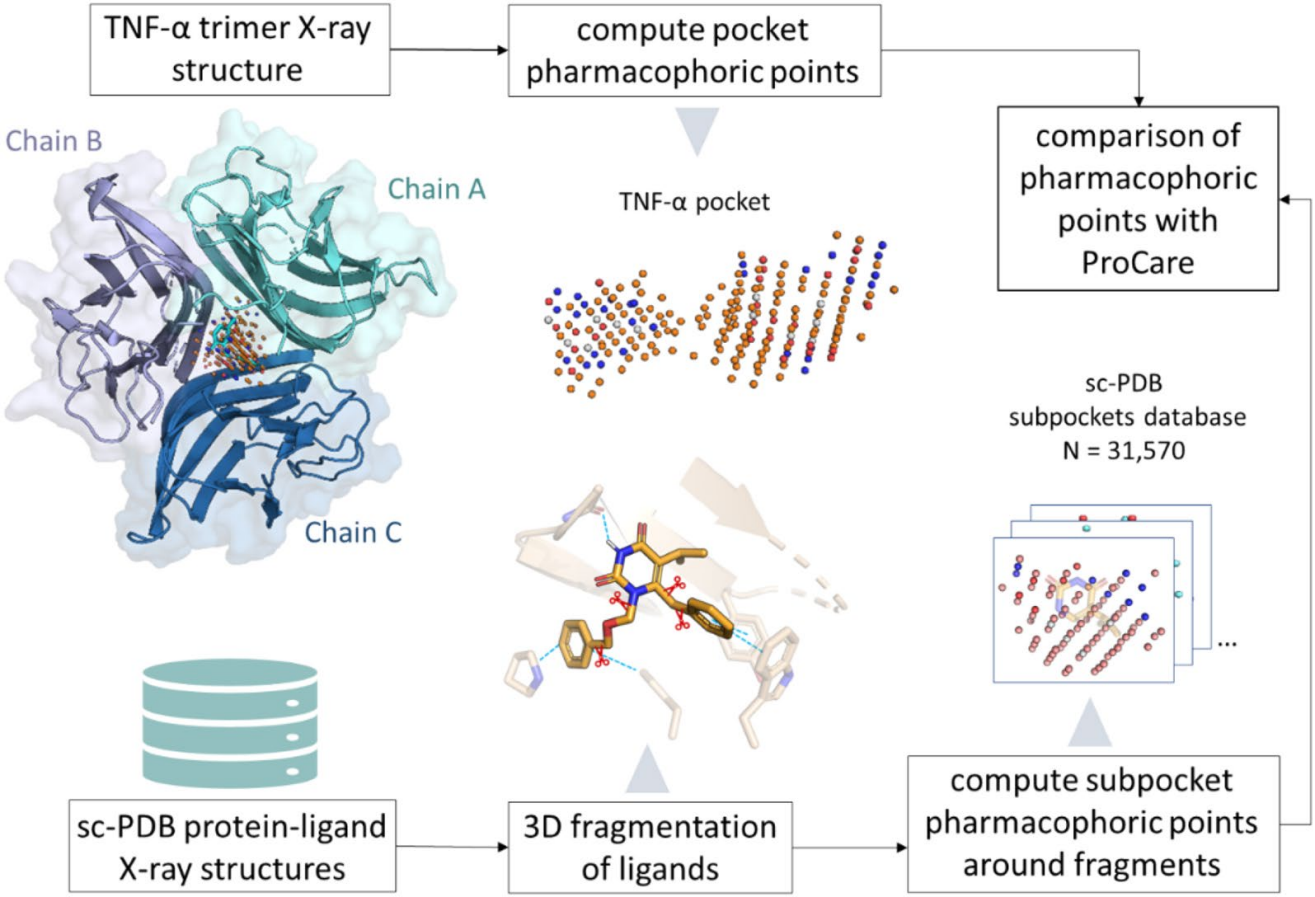
Virtual screening of sc-PDB subpockets for similarity to the core cavity TNF-α. The inner pocket of TNF-α (PDB ID 6OOY) is converted as a cloud of points with pharmacophoric properties (orange: hydrophobic and aromatic, blue: H-bond donor and positive ionizable, red: H-bond acceptor, H-bond donor and acceptor, and negative ionizable, white: dummy) and compared to the corresponding point clouds originating from fragment-bound subpockets of sc-PDB ligands

Identifying similarity between pockets from different proteins suggests that the latter might bind to similar molecules. Although molecular recognition is a dynamic and complex process, the above hypothesis is worth investigating in drug design for hit discovery or for potential off-targets detection. We previously described ProCare [17], a novel computational method relying on a point cloud registration algorithm [19, 20] to assess the similarity between protein pockets. ProCare computes and uses local descriptors, which makes it particularly suitable for detecting local similarities among cavities of different sizes. Typically, ProCare aligns the cavities, described by a cloud of 3D points labeled with pharmacophoric features, by comparing the point descriptors and then derives a similarity score. In the current study (see flowchart in Fig. 1), ProCare was used to detect local similarities between the full cavity of the target protein (here the inner core of the TNF-α trimer) and a collection of 31,570 subpockets from the sc-PDB dataset [8], a repository of 16,034 protein–ligand complexes of known three-dimensional structure for which the ligand is a pharmacological agent bound to a druggable cavity. First, the full cavity of the target protein is computed with the in-house VolSite algorithm [21] and represented by a cloud of pharmacophore-annotated points (Fig. 1). In parallel, the collection of subpocket point clouds is generated after fragmentation of each protein-bound sc-PDB ligand and consideration of the immediate vicinity (4 Å) of generated fragments. Last, the ProCare method aligns and computes the pairwise similarity between the target point cloud, and that from subpockets from the sc-PDB archive (Fig. 1). When a statistically significant similarity is found between a subpocket and the target cavity, the transformation matrix used for the previous alignment is then applied to the corresponding and hidden bound fragment that is directly positioned in the target cavity. In absence of major clashes, the corresponding fragment can therefore be used for a fragment growing or linking strategy or even directly tested for binding to the target.

While benchmarking the ProCare method, we noticed unexpected high similarities (ProCare score > 0.47; p-value < 0.05) between the core pocket at the interface of an inhibitor-bound asymmetric human TNF-α trimer (PDB ID 6OOY) [22], and several non-nucleoside binding sites of inhibitor-bound HIV-1 RT (Additional file 1: Table S1). Notably, seven subpockets from the HIV-1 RT were ranked among the 100 top scoring subpockets, with high ProCare similarity scores (ranging from 0.67 to 0.72) corresponding to very low p-values (from 2.5 × 10^–4^ to 2.1 × 10^–5^).

To assess that the predicted similarity between these unrelated binding sites was not fortuitous, we computed the Receiver-Operating Characteristic (ROC) curve of a binary classifier for which all cavities of a single sc-PDB target (Table 1) are artificially annotated as positives, the rest being defined as negatives. For each target, the ROC curve was defined from the full list of sorted ProCare similarity scores by plotting the true positive rate versus the false positive rate at different threshold settings (Additional file 1: Fig. S1). The area under the ROC curve (ROCAUC) provides a statistical estimation of the accuracy of the classifier to discriminate positives from negatives and therefore predict whether the samples from one particular target are similar (or not) to the TNF-α cavity (Table 1).

**Table 1:** Area under the ROC curve of pairwise ProCare similarity scores^a^

Target	Site	Number of subpockets^b^	ROCAUC
HIV-1 RT	Non-nucleoside	195 (122)	0.84
β2 adrenergic receptor	Orthosteric	14 (14)	0.35
Carbonic anhydrase II	Catalytic	183 (137)	0.38
Cyclin-dependent kinase 2	Catalytic	461 (274)	0.63
Heat shock protein 90α	Catalytic	214 (117)	0.64
Thrombin	Catalytic	253 (126)	0.35

^a^For each target, the similarity scores of the corresponding subpockets (actives) and decoys (any other subpocket) to the TNF-α query (PDB ID 6OOY) are used to compute the area under the ROC curve

^b^Total number of subpockets for the corresponding target. The number of PDB entries are in brackets

Making the hypothesis that the HIV-1 RT non-nucleoside binding pocket is similar to that of TNF-α, the ProCare score nicely discriminates positives (HIV-1 RT) from decoys (all other sc-PDB cavities) with a ROCAUC value (0.84) well above the threshold corresponding to a random classification, ROCAUC = 0.50). Repeating the same exercise with five randomly picked targets (β2 adrenergic receptor, carbonic anhydrase II, cyclin-dependent kinase 2, heat shock protein 90α, and thrombin) lead to much poorer ROC AUC values close or even inferior to random classifications (Table 1). To further exclude a potential bias in the ProCare alignment/scoring method due to the reference TNF-α structure (PDB ID 6OOY) and give a stronger statistical support to our prediction, we systematically compared two additional binding sites (PDB IDs 6OOZ, 6OP0) from available asymmetric human TNF-α X-ray structures [22] to that of 122 HIV-1 RT structures bound to non-nucleoside inhibitors.

### Exhaustive comparison of TNF-α trimer and HIV-1 reverse transcriptase binding sites

**Fig. 2:**
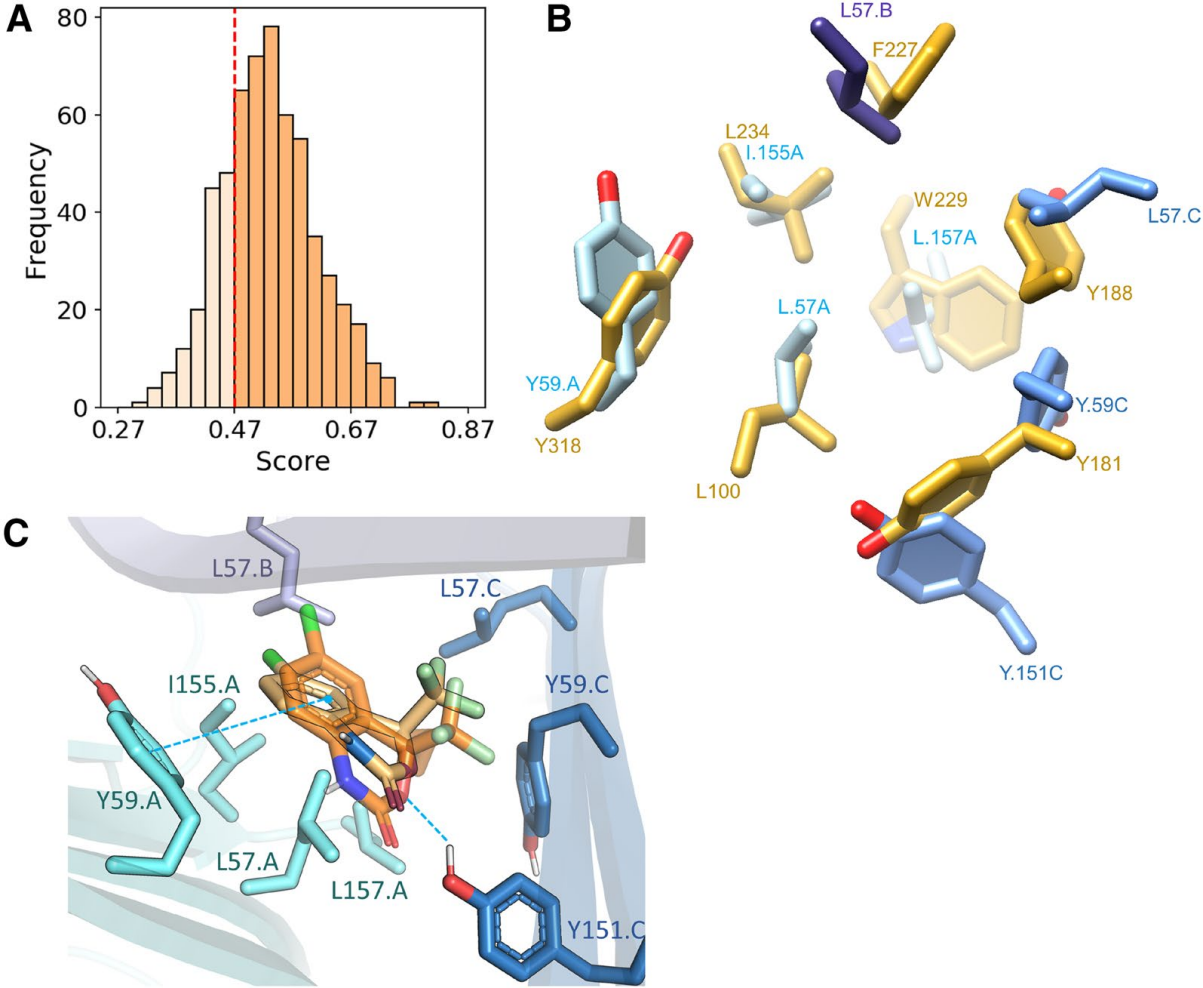
Comparison of TNF-α trimer and HIV-1 RT binding sites with ProCare. **A** Distribution of pairwise similarity scores (n = 195 × 3). Entries scoring above 0.47 (p-value = 0.05; threshold marked by the red dashed line) are considered similar according to a previous statistical analysis of 2 million pairwise alignments [17]. **B** Aligned residues of TNF-α (chain A: cyan, chain B: dark slate blue, chain C: cornflower blue; PDB code: 6OOZ) to HIV-1 RT (orange, PDB code: 1FKO) after rotation and translation of HIV-1 RT protein with the resulting ProCare alignment matrix. **C** ProCare alignment of efavirenz main fragment (light orange) in the TNF-α trimer pocket and PLANTS docking (transparent orange) in the TNF-α trimer pocket (PDB code: 6OOZ). Edge-to-face aromatic interaction with TYR59 of TNF-α chain A and hydrogen bond with TYR151 of TNF-α chain C are depicted by blue dashed lines

A ProCare similarity matrix was built by comparing cavities of three asymmetric TNF-α structures (PDB identifiers 6OOY, 6OOZ and 6OP0) co-crystallized with benzimidazole inhibitors to the 195 subpockets from 122 non-nucleoside HIV-1 RT inhibitors binding sites (Additional file 1: Table S2; Fig. 2) available in the sc-PDB. We observed that 76% of all pairwise comparisons were scored higher than the previously statistically determined ProCare similarity score threshold of 0.47 [17] (Fig. 2A).

**Fig. 3:**
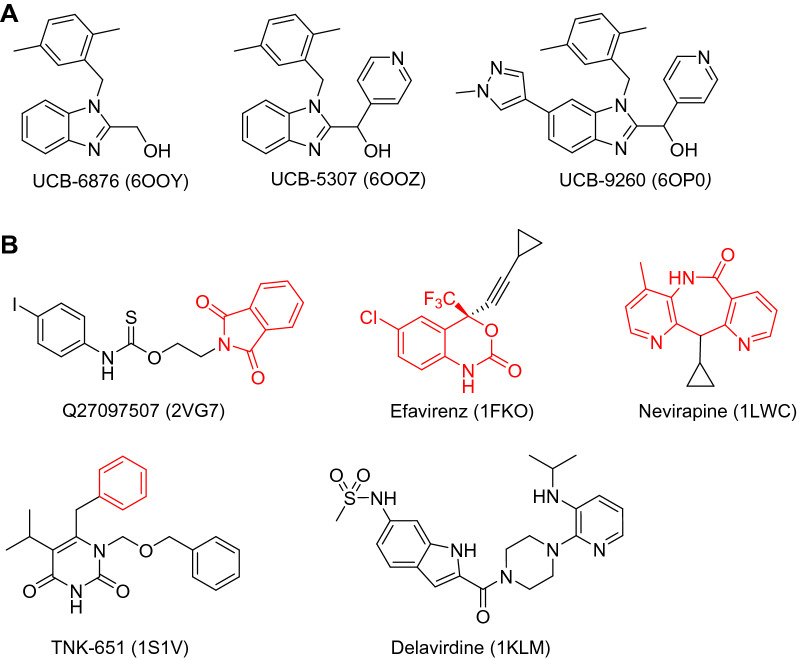
Structures of TNF-α and HIV-1 RT non-nucleoside inhibitors. **A** TNF-α inhibitors and **B** HIV-1 RT non-nucleoside inhibitors (PDB entries between brackets). Red substructures indicate the main fragment binding to the HIV-1 RT subpocket found similar to the TNF-α cavity

To exclude the possibility that the predicted similarity is caused by peculiar mutations of the HIV-1 RT non-nucleoside biding site, we also compared pairwise similarities for both wild type and mutated HIV-1 RT pockets, but did not observe significant differences in the percentage of HIV-1 RT pockets predicted similar to that of TNF-α (74% and 82% of similar pockets for wild type and mutants, respectively). We thus conclude that the predicted similarity between pockets from these two unrelated targets is independent on the chosen PDB structures and is not biased by mutations in the HIV-1 RT binding site. Since ProCare yields a transformation matrix to align the compared objects (subpockets onto the target pockets), we herein provided the visual analysis for one entry (efavirenz-bound subpocket) aligned to the TNF-α structure 6OOZ. Pairs of residues of equivalent interaction properties (aromatic, hydrogen bond donor and acceptor, hydrophobic), respectively in TNF-α and HIV-1 RT were nicely matched (Fig. 2B) demonstrating that the similarity caught with the point clouds is truly present at the residue level. Matched TNF-α/HIV-1 RT residues were: LEU57.A/LEU100; TYR59.A/TYR318; ILE155.A/LEU234; LEU157.A/TRP229; LEU57.B/PHE227; LEU57.C/TYR188; TYR59.C/TYR181 and TYR151.C/TYR181. Inspection of the matched pharmacophoric points that are contributing to the ProCare score showed a mixed contribution of aromatic, hydrogen bond donor and hydrophobic points (Additional file 1: Fig. S2) in agreement with the aligned residues (Fig. 2B) and the statistics of the contributions of the eight pharmacophoric features to the detected similarity (Additional file 1: Fig. S3). Furthermore, efavirenz was docked into TNF-α binding site 6OOZ with PLANTS [26] after validation of the docking protocol by self-docking of the cocrystallized ligand UCB-5307 in 6OOZ (RMSD of top-ranked pose by ChemPLP to crystal coordinates: 0.47 Å, ChemPLP score of -124.79). The ProCare-aligned efavirenz fragment (Fig. 3B) in TNF-α fitted well with one of the PLANTS docking solutions (ranked 3rd/10 with a ChemPLP score of -79.32), corresponding to a RMSD of 1.8 Å of efavirenz main fragment heavy atoms to the ProCare pose (Fig. 2C). Aside the potential hydrophobic interactions in the TNF-α binding site, efavirenz docking pose displayed an edge-to-face aromatic interaction with residue TYR59.A and a hydrogen bond with TYR151.C. Interestingly, efavirenz bound to HIV-1 RT protein structure (1FKO) exhibits an edge-to-face aromatic interaction with residue TYR318 [27] (Additional file 1: Fig. S4A) that was matched by ProCare to TYR59.A in TNF-α (Fig. 2B). Both TYR59.A and TYR151.C are key residues [22] involved in the micromolar and nanomolar binding of the co-crystallized ligands UCB-6876, UCB-5307 and UCB-9260 (Fig. 3) in the TNF-α structures 6OOY, 6OOZ, 6OP0; the interaction between TYR151.C residue and the benzimidazole moiety being a hydrogen bond (Additional file 1: Fig. S4B). Altogether, these observations are strongly suggesting that subpockets in the non-nucleoside binding site of HIV-1 RT are similar to the TNF-α trimer cavity.

Assuming that similar binding sites should accommodate similar ligands, HIV-1 RT non-nucleoside inhibitors should therefore bind to TNF-α. In order to prioritize HIV-1 RT inhibitors for experimental validation of our hypothesis, we checked which inhibitors were bound to the HIV-RT subpockets that are predicted by ProCare as the most similar to the TNF-α cavity (Table 2).

**Table 2:** Bound inhibitors of the HIV-1 reverse transcriptase cavities found similar to TNF-α cavities

HIV-RT inhibitor^a^	HIV1-RT PDB entry	TNF-α PDB entry	ProCare score	Rank
NNI (Q27097507)	2VG7	6OOZ	0.810	1
EFZ (Efavirenz)	1FKO	6OOZ	0.773	2
NVP (Nevirapine)	1LWC	6OOZ	0.737	3
TNK (TNK-651)	1S1V	6OOZ	0.731	4
NVP (Nevirapine)	2HNY	6OOZ	0.729	5
…		…	…	…
SPP (Delavirdine)^b^	1KLM	6OOZ	0.484	408

^a^PDB chemical component identifier (Name in brackets)

^b^After manual fragmentation, a higher ProCare score (0.599) was obtained for the subpocket of delavirdine’s fragment #2 (Additional file 1: Fig. S5) against 6OOY pocket (Additional file 1: Table S3)

Among the corresponding inhibitors, two compounds (Q27097507, TNK6-51) are not commercially available and were not considered. However, two easily purchasable FDA-approved drugs (efavirenz, nevirapine; Fig. 3) are almost entirely buried in the HIV-1 RT subpockets found similar to the TNF-α cavity, exhibit a size and molecular volume similar to that of two TNF-α inhibitors (UCB-6876 and UCB-5307; Fig. 3) and were therefore selected for biological evaluation. In addition, we also considered a third marketed inhibitor (delavirdine; Table 2, Fig. 3) whose pocket was found much less similar to that of TNF-α, although just above the 0.47 ProCare similarity threshold.

### Non-nucleoside HIV-1 RT inhibitors bind to human TNF-α

**Fig. 4:**
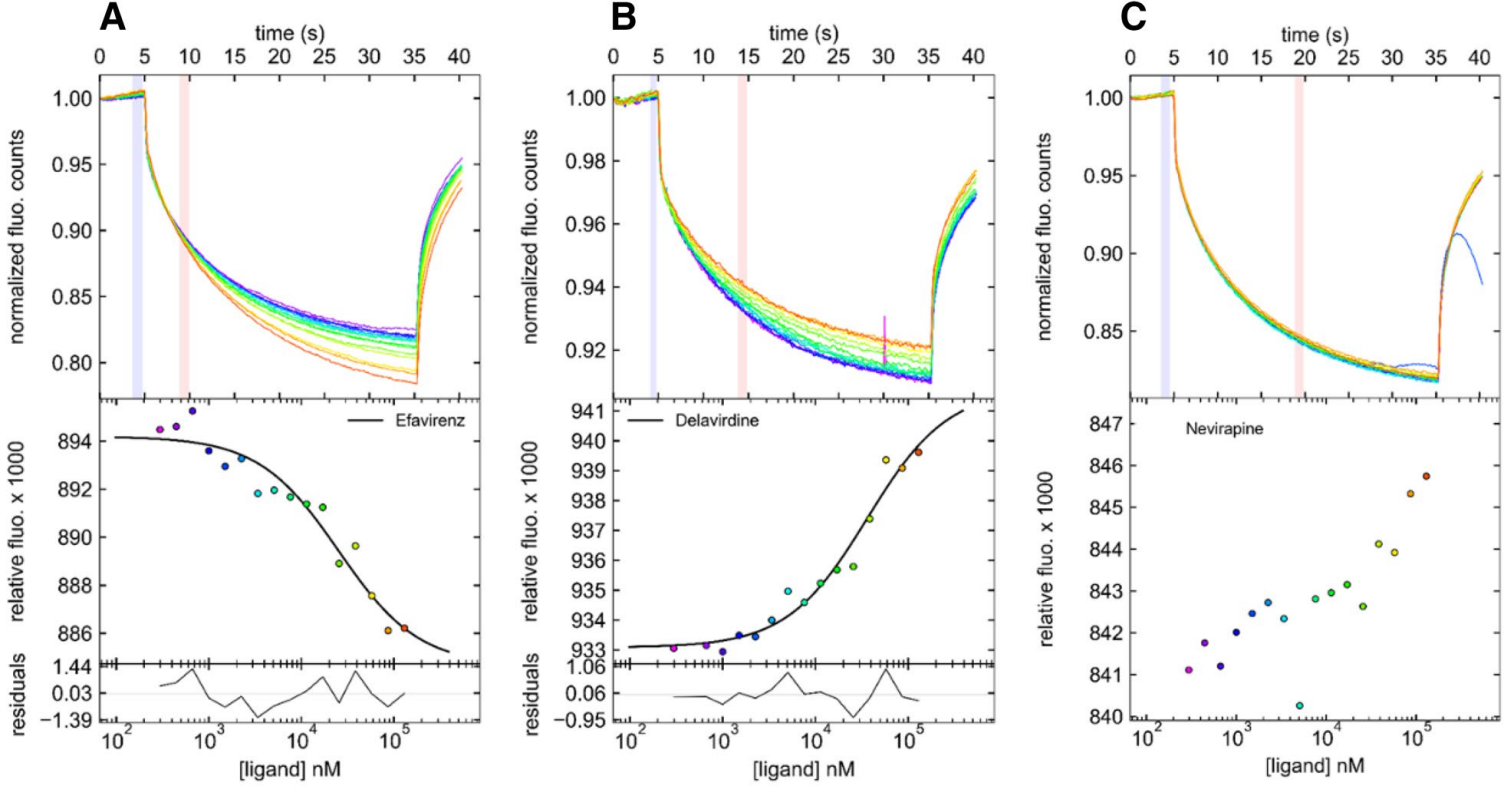
Microscale thermophoresis (MST) demonstrates a direct interaction between HIV-1 RT inhibitors and RED fluorescent-tagged TNF-α. For analysis, the change in thermophoresis is expressed as the change in the normalized fluorescence (Δ*F*_norm_), which is defined as *F*_hot_/*F*_cold_ (*F*-values correspond to average fluorescence values between defined areas marked by the red and blue cursors). Titration of the non-fluorescent ligand results in a gradual change in thermophoresis, which is plotted as Δ*F*_norm_ to yield a binding curve, which can be fitted to derive binding constants. **A** Experimental MST traces of efavirenz (*K*_D_ = 24 ± 8 µM); **B** Experimental MST traces of delavirdine (*K*_D_ = 39 ± 9 µM); **C** Experimental MST traces of nevirapine. Only the best MST traces (highest signal to noise ratio) are shown here. Values for all experiments conducted according to different experimental protocols are listed in Additional file 1: Table S4

Three different non-nucleoside FDA-approved drugs (nevirapine, efavirenz and delavirdine) were tested for direct binding to a fluorescent-labelled TNF-α trimer by microscale thermophoresis (MST), a robust and sensitive biophysical method to detect and quantify molecular interactions in solution [28, 29]. The MST signal is based on ligand-dependent temperature-induced changes (thermophoresis, temperature-related fluorescence intensity) of the fluorescence emission of the labelled protein target. The 17.3 kDa homotrimeric TNF-α that spontaneously assembles in solution [30, 31] was therefore labelled by a RED-fluorescent probe for MST experiments in presence of increasing concentrations of the three HIV-1 RT inhibitors (Fig. 4).

MST traces in presence of efavirenz and delavirdine showed distinct states (from bound to unbound), indicating a direct interaction of these two inhibitors with TNF-α (Fig. 4A, B). Dissociation constants (*K*_D_) could be derived for the two corresponding complexes and estimated to 24 ± 8 µM (Efavirenz) and 39 ± 9 µM (Delavirdine), respectively (Fig. 4A, B). The measured dissociation constants for the two HIV-1 RT inhibitors are in the same range of magnitude than that of UCB-6876 (K_D_ = 22 µM) [22], one of the three TNF-α inhibitors used as a reference for this study.

Contrarily to our prediction, no thermophoresis signal could be detected in presence of nevirapine (Fig. 4C) indicating no binding of this inhibitor to TNF-α, at least in our experimental settings. The herein observations were insensitive to experimental protocols (buffer composition, solubilizing agents, incubation time, MST power; Additional file 1: Table S4).

In absence of X-ray structures of TNF-α bound to efavirenz and delavirdine, we cannot rule out the possibility that both inhibitors bind to a different pocket than that highlighted in the current computational study. This hypothesis is however very unlikely for two reasons: (i) no other cavity than that occurring at the inner core of the multimeric TNF-α could be detected among the currently existing 33 structures available in the Protein Data Bank; (ii) all non-covalent small molecular weight inhibitors co-crystallized with TNF-α dimeric or trimeric forms [32–35] are exactly bound at the central pocket examined in this study.

We should recall here that none of the HIV-1 RT inhibitors has been optimized for binding to TNF-α and is directly repurposable for treating TNF-α -dependent autoimmune disorders. However, we do think that efavirenz may be optimized to a much more potent HIV-1 RT inhibitor by following a strategy similar to that reported to modify the 22 µM TNF-α inhibitor UCB-6876 to a 9 nM lead (UCB-5307; Fig. 3) by just occupying a side pocket formed by the three TYR199 side chains of the TNF-α homotrimer with a pyridyl ring [22]. Structure-guided efavirenz optimization for TNF-α binding is therefore possible by appropriate trimming of unnecessary cyclopropylethynyl substituent and occupation of the above-described potency subpocket.

### The similarity between TNF-α trimer and HIV-1 reverse transcriptase binding sites is not obvious

**Fig. 5:**
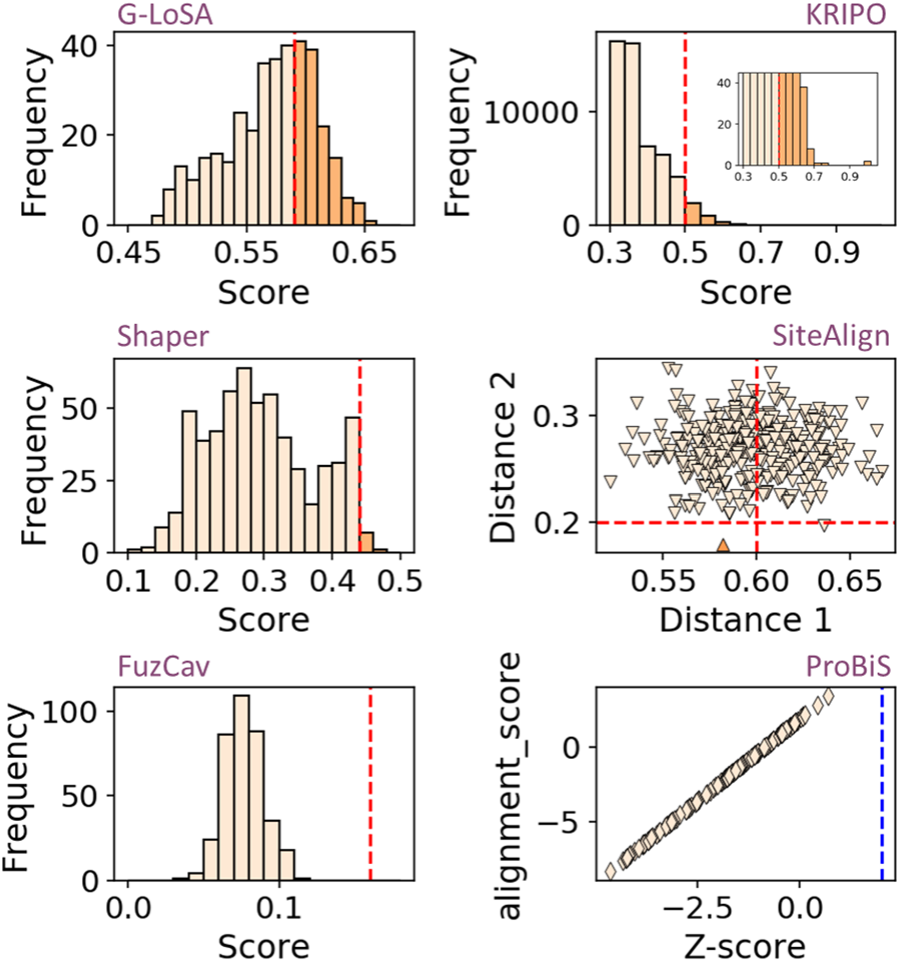
Score distribution of pairwise comparisons between binding sites of TNF-α trimer and HIV-1 reverse transcriptase. Binding sites in asymmetric structures of TNF-α trimer (n = 3) were compared to binding sites of non-nucleoside inhibitors in HIV-1 reverse transcriptase (sc-PDB set, n = 122). Pairs with similarity measures scored above each method-specific threshold (red dashed line) were considered similar. For SiteAlign comparisons, pairs are considered similar in case the two distances (distance 1, distance 2) are below the recommended cut-off. For ProBiS, the threshold above which an alignment is considered significant is marked by the blue dashed line

To demonstrate whether the herein disclosed similarity between the human TNF-α trimer and the HIV-1 RT non-nucleoside binding sites is obvious, we performed the same set of pairwise binding site comparisons, as that previously reported for ProCare (Fig. 2), with state-of-the-art methods [10] developed either in-house (FuzCav [36], Shaper [21] and SiteAlign [37]) or by third parties (G-LoSA [38], KRIPO [15] and ProBiS [39]). The binding site perception, comparison algorithm and scoring function is specific to each method. Some methods (FuzCav, SiteAlign) consider entire cavities while some others utilize either fragment-bound subpockets (KRIPO, Shaper) or local protein descriptors (G-LoSA). To make the comparison consistent, the same set of atomic coordinates were compared, a binding site being defined by the protein PDB identifier, the ligand PDB HET record (three alphanumeric character describing non-standard PDB residues), chain identifiers and list of amino acids lining the cavity. The only exception was for the KRIPO method, which used all the chains available in the PDB entry, but still corresponding to the same tuple (PDB, HET) as for the other methods. For each method, the distribution (Fig. 5) and percentage of pairwise comparisons scored above the developer’s recommended similarity threshold (Table 3) were reported.

**Table 3:** Comparison of three TNF-α and 122 HIV-1 RT non-nucleoside binding sites by state-of-the-art cavity comparison methods

Method	Score threshold^a^	Metric	Success rate^b^
G-LoSA	0.59	GA-score	35.2
KRIPO	0.50	Modified Tanimoto coefficient	5.8
Shaper	0.44	ColorRefTversky	1.4
SiteAlign	0.6, 0.2	d1 and d2 distances^c^	0.3
FuzCav	0.16	Tanimoto coefficient	0
ProBiS	2	Z-score^d^	0
ProCare	0.47	ProCare score	76.6

^a^Developer’s recommended similarity/distance threshold for estimating two binding sites similar

^b^Percentage of pairwise comparisons scored above the threshold

^c^For SiteAlign comparisons, pairs are considered similar when the two distances (d1, d2) are below the score threshold value [37]

^d^The Z-score indicates the statistical relevance of ProBiS binding site alignments

Strikingly, only the G-LoSA method relying on a graph-based local alignment of cavity-lining amino acids, managed to find some similarity between the two sets of binding sites, however with reduced success rate (35.2%) when compared to the ProCare algorithm (76.6% success rate; Table 3). We acknowledge that the developer's recommended thresholds may be biased toward peculiar datasets. However, all methods compared herein were subjected to the same protocol and we do think that the threshold scores are appropriate indicators in a virtual screening setting where there is no room for a one-by-one case study of each pairwise comparison.

**Fig. 6:**
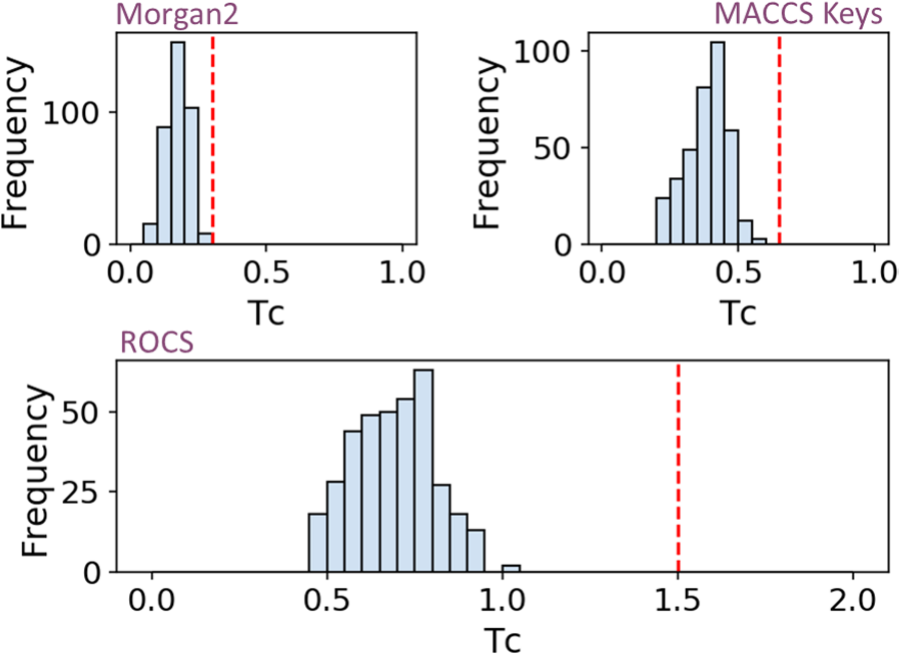
Pairwise similarity between inhibitors of TNF-α trimer and non-nucleoside inhibitors of HIV-1 reverse transcriptase. Recently described TNF-α trimer inhibitors (n = 3) were compared to non-nucleoside inhibitors of HIV-1 RT (sc-PDB set, n = 122). Pairs with similarity measures scored above each descriptor-specific threshold (red dashed line) were considered similar. (Top left) 2D similarity estimated by a Tanimoto metric using Morgan2 circular fingerprint, (Top right) 2D similarity estimated by a Tanimoto metric using 166 MACCS public keys. (Bottom) 3D shape comparison (ROCS) estimated by the TanimotoCombo metric

The herein reported binding of some HIV-1 RT non-nucleoside inhibitors to human TNF-α remains unobvious to many binding site comparison algorithms. Would this unexpected feature be better captured by remote ligand similarities? To investigate this question, we compared 2D and 3D descriptors of the corresponding inhibitors (Fig. 6).

Neither comparing 2D fingerprints nor 3D shapes would have confidently suggested possible binding of HIV-1 RT inhibitors to TNF-α trimer (Fig. 6) since none of the considered ligand pairs exhibit a pairwise similarity above an acceptable threshold (Morgan2 circular fingerprint: 0.30 [40]; 166 public MACCS keys: 0.65 [40], TanimotoCombo ROCS 3D similarity: 1.5 [41, 42]). We should precise here that 3D similarities were inferred from PDB protein-bound ligand X-ray structures and that alternative conformations might be selected by the two targets, although the very rigid efavirenz does indeed bind to the two proteins of interest albeit with different affinities (TNF-α, K_D_ = 24 μM; HIV-1RT, ChEMBL median IC_50_ = 20 nM). Extending 2D fingerprint comparisons to additional 2,361 HIV-1 RT inhibitors (Additional file 1: Table S5) from the ChEMBL database [43], did not change our conclusion since only 0.71% and 0.09% of the corresponding pairs were found similar using Morgan2 and 166 public MACCS keys, respectively (data not shown).

## Conclusion

Herein, we describe a systematic comparison of fragment-bound subpockets from a priori unrelated targets (TNF-α, HIV-1 RT) but predicted to share local similarities according to our recently-developed ProCare point cloud registration method. The computational prediction was verified by microscale thermophoresis experiments evidencing the micromolar binding of some but not all HIV-1 RT non-nucleoside inhibitors to human soluble TNF-α. Interestingly, the ProCare prediction could not be revealed by other state-of-the-art cavity or ligand similarity search methods. Point cloud registration, a computational method frequently used for digital image processing in robotics and medical imaging, enables the detection of subtle and local protein similarities thanks to a powerful description of subpocket microenvironments. The ProCare method appears as a promising idea generator for drug repurposing and fragment-based ligand design since it is able to pick starting ligands at a proteomic scale.

## Methods

### Preparation of protein and ligand structures

#### TNF-α structures

The recently described asymmetric structures of the human TNF-α trimer bound to different inhibitors were retrieved from the RCSB Protein Data Bank (PDB) homepage (https://www.rcsb.org) [44] using the following identifiers: 6OOY, 6OP0, 6OOZ [22]. The PDB structures were protonated with Protoss [45] v.4.0, then split into protein, ligands and water molecules and finally converted into mol2 format with Sybyl-X v.2.1.1 (Certara USA, Inc., Princeton, NJ 08540). The binding sites (‘SITE’) were defined as any protein residue with at least one heavy atom closer than 6.5 Å from any ligand heavy atom and saved in mol2 and pdb formats. The ligands were converted into sdf format with OpenEye Python toolkits v.2020.0.4 (OpenEye Scientific Software, Santa Fe, USA). Cavities were detected with IChem v.5.2.9 VolSite utility [21] (cavity_all output) using default parameters. The cavity points are labeled with eight possible pharmacophoric features (hydrophobic, aromatic, H-bond donor, H-bond acceptor, H-bond donor and acceptor, positive, negative, dummy) that are complementary to the features of the nearest protein atom. If no protein atom is found within a 4 Å distance of a cavity point, the latter is assigned a dummy property.

#### HIV-1 reverse transcriptase PDB structures

Starting from the PDB structure 1VRT as a reference, a search was performed in the RCSB PDB (https://www.rcsb.org) [44] to retrieve all structures with strict matching (“Structure Similarity” query in the PDB). After visual check, 122 entries already available in the sc-PDB repository (http://bioinfo-pharma.u-strasbg.fr/scPDB) [8] and for which the ligand is a non-nucleoside inhibitor were kept. The remaining PDB structures were protonated with Protoss [45] v4.0. The list of the PDB identifiers and Uniprot accession numbers is reported Additional file 1: Table S2. According to the sc-PDB preparation rules, the binding sites (‘SITE’) were defined as described above. Protein, ligand and binding site ‘SITE’ structures were directly retrieved in mol2 file format from the sc-PDB archive. The corresponding 122 ligands were 3D-fragmented with the IChem v.5.2.9 [49] fragmentation utility [47] and the complementary VolSite [21] cavity points, computed at 4 Å around each fragment were finally saved. The ligands were converted into sdf format as described above.

#### Preparation of HIV-1 reverse transcriptase ChEMBL ligands

Bioassay information were first retrieved from the ChEMBL [43] dataset (Release 28; https://www.ebi.ac.uk/chembl) by querying the general keyword ‘reverse transcriptase’ and retaining ChEMBL target identifiers (CHEMBL247, CHEMBL4296301, CHEMBL2366516) corresponding to HIV-1 RT. Ligands with a measured sub-micromolar half-maximal inhibitory concentration (IC50) against the HIV1-RT single target were defined here as inhibitors (Additional file 1: Table S5). The corresponding SMILES strings were retrieved and further processed with RDKit (Open-source cheminformatics; http://www.rdkit.org) v.2019.03.4.0 to remove redundancy.

#### Preparation of sc-PDB fragments and subpockets

Ligands coordinates from the sc-PDB (http://bioinfo-pharma.u-strasbg.fr/scPDB) [46] v.2016 archive were fragmented in 3D with the IChem v.5.2.9 fragmentation utility [47]. Fragmentations occur in the binding sites so that only the main fragments interacting sufficiently (four interactions of which at least one is polar) with their target proteins were kept. Finally, the cavity pharmacophoric points cloud were computed at 4 Å from the fragments center to describe the protein subpocket, using the IChem v.5.2.9 VolSite utility (“cavity_4” output). VolSite cavities exhibiting less than three points were removed. A total of 31,570 valid fragment-bound subpockets were finally obtained.

### Cavity similarities

#### ProCare

ProCare [17] v.0.1.1 pairwise comparison were performed on cavities computed with the VolSite module [21] in IChem v5.2.9 [49]. Entire cavities (“cavity_all” output) were calculated for TNF-α structures whereas only cavity points closer than 4.0 Å from any fragmented ligand center (“cavity_4” output) were considered for sc-PDB subpockets. VolSite cavity points were directly used for point cloud registration starting with determination of colored fast point feature histograms (c-FPFH) as previously described [17]. Finally, the respective set of c-FPFH descriptors for the two cavities were compared to each other using a RANSAC algorithm [19, 20] followed by refinement with default parameters [17]. Alignments results were scored with the default ProCare scoring function [17] which evaluates with a Tversky metric the proportion of aligned points of the same pharmacophoric features. In agreement with our previous study [17] where the similarity threshold of 0.47 (p-value of 0.05) was statistically determined, pockets scoring above 0.47 were considered similar.

#### FuzCav

FuzCav [36], an alignment-free method, was used to compare the binding site ‘SITE’ (mol2 format) entries of TNF-α dataset to the binding sites of HIV-1 RT sc-PDB dataset. Each binding site was tagged to compute a 4,833 bit-string that count all possible pharmacophoric triplets based on the atomic coordinates of Cα atoms lining the binding cavity. The pairwise comparisons of the fingerprints were evaluated with the default similarity score, with a threshold set at a value of 0.16 to distinguish similar from dissimilar binding sites.

#### G-LoSA

G-LoSA [38] v.2.2 is an alignment tool that was used with the binding sites ‘SITE’ pdb files. G-LoSA computes a set of inter-structural Cα pair distances to derive a graph, which will later be subjected to maximum clique search. The default G-LoSA score (GA-score) was used to evaluate the alignments. A threshold value of 0.59, recommended by the authors [38] and corresponding to a p-value of 0.05, was used to distinguish similar from dissimilar binding sites.

#### KRIPO

PDB ligands structural information were downloaded from Ligand Expo (http://ligand-expo.rcsb.org/) and prepared according to the KRIPO procedure (https://github.com/3D-e-Chem/kripo). Then KRIPO [15] v.1.0.1 was used with the list of prepared PDB structures for the pharmacophore fuzzy fingerprints calculations, using default parameters (fragmentation procedure activated). The pairwise similarities of the fingerprints were estimated with kripodb (v.3.0.0) using the modified Tanimoto coefficient as similarity metric. A threshold value of 0.50 was used to distinguish similar from dissimilar binding sites.

#### ProBiS

In a first place, the surface information (srf files) was computed for each prepared PDB structures with the default parameters referenced in the manual (3.0 Å to the ligand). ProBiS [39] requires a list of ligand HET code and residue number for each PDB entries. That list was provided to ensure that the ligands/sites considered are the same as in the binding site datasets used for other methods. Then, the alignment and comparison of the srf files were executed with default parameters, except for the Z-score that was set to a high negative value (− 9999) as suggested by the authors to enforce the output of all results. Similarity between two binding sites was evaluated by the alignment score and Z- score. A threshold Z-score value of 2.0 was used to distinguish significant from irrelevant binding site alignments.

#### SiteAlign

For each entry, the list of natural amino acids in the ‘SITE’ mol2 files were provided as input. SiteAlign [37] v.4.0 describes a binding site by a polyhedron of 80 discretized triangles annotated with eight possible pharmacophoric features projected from cavity-lining C-α atoms. This results in a fingerprint of 640 integers. The pairwise comparisons imply aligning the corresponding polyhedron and computing the d1 and d2 distances of the fingerprints. The distance thresholds of d1 = 0.6 and d2 = 0.2 were applied respectively, to discriminate similar from dissimilar binding sites.

#### Shaper

Shaper [21] v.1.0 uses the same input files (VolSite cavities in mol2 file format) as ProCare. Shaper is an alignment method based on the OpenEye ShapeTK toolkit (OpenEye Toolkits 2020.2.0, OpenEye Scientific Software, Santa Fe, USA) to maximize the overlap of shape and pharmacophoric features of the two compared cavities, thanks to a smooth Gaussian function. The alignments were realized with default settings and scored with a Tversky metric putting more weight on the reference cavity (RefTve). A threshold RefTve value of 0.44 (p-value = 0.005) was used to distinguish similar from dissimilar binding sites.

### Ligand similarities

#### Ligand 2D similarity

Morgan fingerprints on the one hand, and 166 public MACSS keys on the other hand were computed on the PDB ligands (sdf format) and ChEMBL ligands (SMILES strings) with RDKit (Open-source cheminformatics; http://www.rdkit.org) python package v.2019.03.4.0 using default parameters (radius = 2 for the Morgan fingerprints). The Tanimoto coefficients of the pairwise TNF-α ligands/HIV-1 RT ligands fingerprints comparison were reported. Cut-off values of 0.30 (Morgan fingerprints) and 0.65 (MACCS keys) were used to discriminate chemically similar from dissimilar ligands.

#### Ligand 3D similarity

sc-PDB HIV-1 RT inhibitors were compared to TNF-α inhibitors with OpenEye ROCS v.3.2.0.4 and scored by decreasing Tanimoto similarity metric accounting for both shape and chemical features overlap (TanimotoCombo). A TanimotoCombo cut-off value of 1.5 was used to discriminate chemically similar from dissimilar ligands.

### Docking

TNF-α X-ray structure 6OOZ was prepared as described above (see TNF-α structures). 6OOZ co-crystallized ligand on the one hand, delavirdine, efavirenz and nevirapine as well as their main fragments on the other hand were drawn with MarvinSketch v.16.10.17 (ChemAxon Ltd, 1031 Budapest, Hungary) and saved into 2D sdf format. They were ionized with Filter v.2.5.1.4 (OpenEye Scientific Software, Santa Fe, USA) using customized parameters (Additional file 1: Table S6). Then Corina v.3.40 (Molecular Networks GmbH, 90411 Nürnberg, Germany) was used to generate a starting 3D conformation for each inhibitor. The prepared molecules were docked into the target 6OOZ with PLANTS v.1.2 [26] using the following configuration: the grid was set at 13 Å from the binding site center; poses were searched ‘speed1’ settings to generate a maximum of 10 poses per ligand using a clustering rmsd of 2 Å. Solutions were scored with the default ChemPLP scoring function [26]. The docking protocol was validated by computing the RMSD of the docked 6OOZ ligand coordinates and the X-ray coordinates. Results were processed and rescored by computing the interaction fingerprint (IFP) similarity (Tanimoto metric) [48] between X-ray and docking poses. The IFPs were computed with IChem v.5.2.9 IFP module. All poses were visually inspected using Maestro v.2019-3 (Schrödinger, New York, NY 10036-4041).

### Chemicals and biologicals

Nevirapine (catalog #S1742), efavirenz (catalog #S4685) and delavirdine mesylate (catalog #S6452) were purchased from Selleck Chemicals (https://www.selleckchem.com/). Soluble human TNF-α (catalog # Z01001) was purchased from GenScript (http://www.genscript.com).

### Binding of HIV-1 RT inhibitors to human TNF-α (microscale thermophoresis)

Human TNF-α was labeled using the RED-NHS 2nd generation labeling kit (NanoTemper Technologies GmbH) using a protein concentration of 10 µM and a molar dye-to-protein ratio ~ 3:1. A label/protein ratio of 0.4 was determined using photometry at 650 and 280 nm. Compounds efavirenz, delavirdine and nevirapine were initially dissolved in DMSO to afford stock solutions of 10 mM. These were then diluted to initial concentrations of 260 μM into 20 mM K-phosphate pH 7.4, 150 mM NaCl ensuring a final concentration of DMSO of 2.6%. These compounds were serially diluted 2:1 in buffer 20 mM K-phosphate pH 7.4, 150 mM NaCl, 2.6% DMSO producing ligand concentrations ranging from 260 µM to 594 nM (16 titration points). For MST measurements, each ligand dilution was mixed with 1 volume of fluorescently-labelled TNF-α at 680 nM in 20 mM K-phosphate pH 7.4, 150 mM NaCl, 0.02% Tween-20, which leads to a final concentration of TNF-α of 340 nM and final ligand concentrations at half of the ranges above. The final buffer is 20 mM K-phosphate pH 7.4, 150 mM NaCl, 0.01% Tween-20 and 1.3% DMSO. After a 15-min incubation at room temperature in the dark, followed by centrifugation at 13,000*g* for 3 min, each solution was filled into Monolith NT Premium capillaries (NanoTemper Technologies GmbH). Thermophoresis was measured at 25 °C with 40% LED power and 20%, 40% and 80% MST power using a Monolith NT.115 (NanoTemper Technologies GmbH). Data were analyzed in the NT Analysis software version 1.5.41 (NanoTemper Technologies GmbH).

## Supplementary Information

**Additional file 1: Fig. S1**: Receiver operating characteristic (ROC) curves derived from ProCare similarity scores. **Fig. S2**: ProCare alignment of efavirenz main fragment subpocket onto TNF-α trimer pocket. **Fig. S3**: Contributions of the eight pharmacophoric features to the ProCare similarity score between HIV-1 RT and TNF-α. **Fig. S4**: Non-covalent interactions between efavirenz and HIV-1 RT, and between UCB-5307 and TNF-α trimer. **Fig. S5**: Manual fragmentation of delavirdine in three fragments (#1 to #3). **Table S1**: sc-PDB subpockets sorted by decreased ProCare similarity to the inner cavity of human TNF-α. **Table S2**: PDB entries describing non-nucleoside inhibitors bound to HIV-1 reverse transcriptase. **Table S3**: Comparison of delavirdine subpockets, resulting from manual fragmentation, with TNF-α trimer pockets. **Table S4**: Dissociation constant (KD) of three HIV-1 RT inhibitor binding to human soluble TNF-α, according to MST experimental conditions. **Table S5**: CHEMBL entries describing HIV-1 RT non-nucleoside inhibitors. **Table S6**: Customized rules for OpenEye Filter ionization.

## Data Availability

*Data*: Input and results data are available at https://github.com/kimeguida/ProCare_TNF. *Software*: ProCare, version 0.1.1 and 0.1.0, https://github.com/kimeguida/ProCare; Fuzcav, http://bioinfo-pharma.u-strasbg.fr/labwebsite/downloads/FuzCav.tgz; G-LoSA, version 2.2, https://compbio.lehigh.edu/GLoSA; KRIPODB, version 3.0.0, http://3d-e-chem.github.io/kripodb; KRIPO, version 1.0.1, https://github.com/3D-e-Chem/kripo; ProBiS, http://insilab.org/probis-algorithm/; SiteAlign, version 4.0, http://bioinfo-pharma.u-strasbg.fr/labwebsite/downloads/SiteAlign-4.0.tgz; Shaper, version 1.0, http://bioinfo-pharma.u-strasbg.fr/labwebsite/downloads/Shaper.tgz; RDKit python package, version 2019.03.4.0, https://www.rdkit.org/; ROCS, version 3.2.0.4, https://www.eyesopen.com/rocs; IChem, version 5.2.9, http://bioinfo-pharma.u-strasbg.fr/labwebsite/downloads/IChem_v.5.2.9.tgz: Python OpenEye toolkits version 2020.0.4; FILTER, version 2.5.1.4, https://www.eyesopen.com/filter; PLANTS version 1.2, http://www.tcd.uni-konstanz.de/plants_download; Python package Matplotlib version 3.0.2; Maestro vesion 2019–3, https://www.schrodinger.com/products/maestro; Pymol version 2.1, https://pymol.org/2; Sybyl-X v.2.1.1, https://www.certara.com/sybyl-x-software; MarvinSketch version 16.10.17, https://chemaxon.com/products/marvin.

## Abbreviations

2D: Two-dimension
3D: Three-dimension
AUC: Area under the curve
c-FPFH: Colored fast point feature histogram
DMSO: Dimethyl sulfoxide
HIV: Human immunodeficiency virus
MST: Microscale thermophoresis
PDB: Protein data bank
RANSAC: Random sample consensus
RMSD: Root mean square deviation
ROC: Receiver operating characteristics
RT: Reverse transcriptase
TNF: Tumor necrosis factor
