# Supplementary material for: Unexpected similarity between HIV-1 reverse transcriptase and tumor necrosis factor binding sites revealed by computer vision
Source: J Cheminform. 2021 Nov 23;13:90. doi: 10.1186/s13321-021-00567-3 (PMC8609734; doi:10.1186/s13321-021-00567-3)
Supplement: Supplementary file 1 — Additional file 1: Fig. S1: Receiver operating characteristic (ROC) curves derived from ProCare similarity scores. Fig. S2: ProCare alignment of efavirenz main fragment subpocket onto TNF-α trimer pocket. Fig. S3: Contributions of the eight pharmacophoric features to the ProCare similarity score between HIV-1 RT and TNF-α. Fig. S4: Non-covalent interactions between efavirenz and HIV-1 RT, and between UCB-5307 and TNF-α trimer. Fig. S5: Manual fragmentation of delavirdine in three fragments (#1 to #3). Table S1: sc-PDB subpockets sorted by decreased ProCare similarity to the inner cavity of human TNF-α. Table S2: PDB entries describing non-nucleoside inhibitors bound to HIV-1 reverse transcriptase. Table S3: Comparison of delavirdine subpockets, resulting from manual fragmentation, with TNF-α trimer pockets. Table S4: Dissociation constant (KD) of three HIV-1 RT inhibitor binding to human soluble TNF-α, according to MST experimental conditions. Table S5: CHEMBL entries describing HIV-1 RT non-nucleoside inhibitors. Table S6: Customized rules for OpenEye Filter ionization. [file 13321_2021_567_MOESM1_ESM.pdf]

## Supplementary information

# Unexpected similarity between HIV-1 reverse transcriptase and tumor necrosis factor binding sites revealed by computer vision.

Merveille Eguida[1] and Didier Rognan[1,*]

[1] Laboratoire d'Innovation Thérapeutique, UMR 7200 CNRS-Université de Strasbourg, 67400 Illkirch, France.

* Corresponding author

E-mail: rognan@unistra.fr

**Figure S1** Receiver operating characteristic (ROC) curves of ProCare similarity scores.

**Figure S2.** ProCare alignment of efavirenz main fragment subpocket onto TNF-α trimer pocket.

**Figure S3.** Contributions of the eight pharmacophoric features to the ProCare similarity score between HIV-1 RT and TNF-α.

**Figure S4.** Non-covalent interactions between efavirenz and HIV-1 RT; and between UCB-5307 and TNF-α trimer.

**Figure S5.** Manual fragmentation of delavirdine in three fragments (#1 to #3).

**Table S1.** sc-PDB subpockets sorted by decreased ProCare similarity to the inner cavity of human TNF-α.

**Table S2.** PDB entries describing non-nucleoside inhibitors bound to HIV-1 reverse transcriptase.

**Table S3.** Comparison of delavirdine subpockets, resulting from manual fragmentation, with TNF-α trimer pockets.

**Table S4.** Dissociation constant ($K_D$) of three HIV-1 RT inhibitor binding to human soluble TNF-α, according to MST experimental conditions.

**Table S5.** CHEMBL entries describing HIV-1 RT non-nucleoside inhibitors.

**Table S6.** Customized rules for OpenEye Filter ionization.

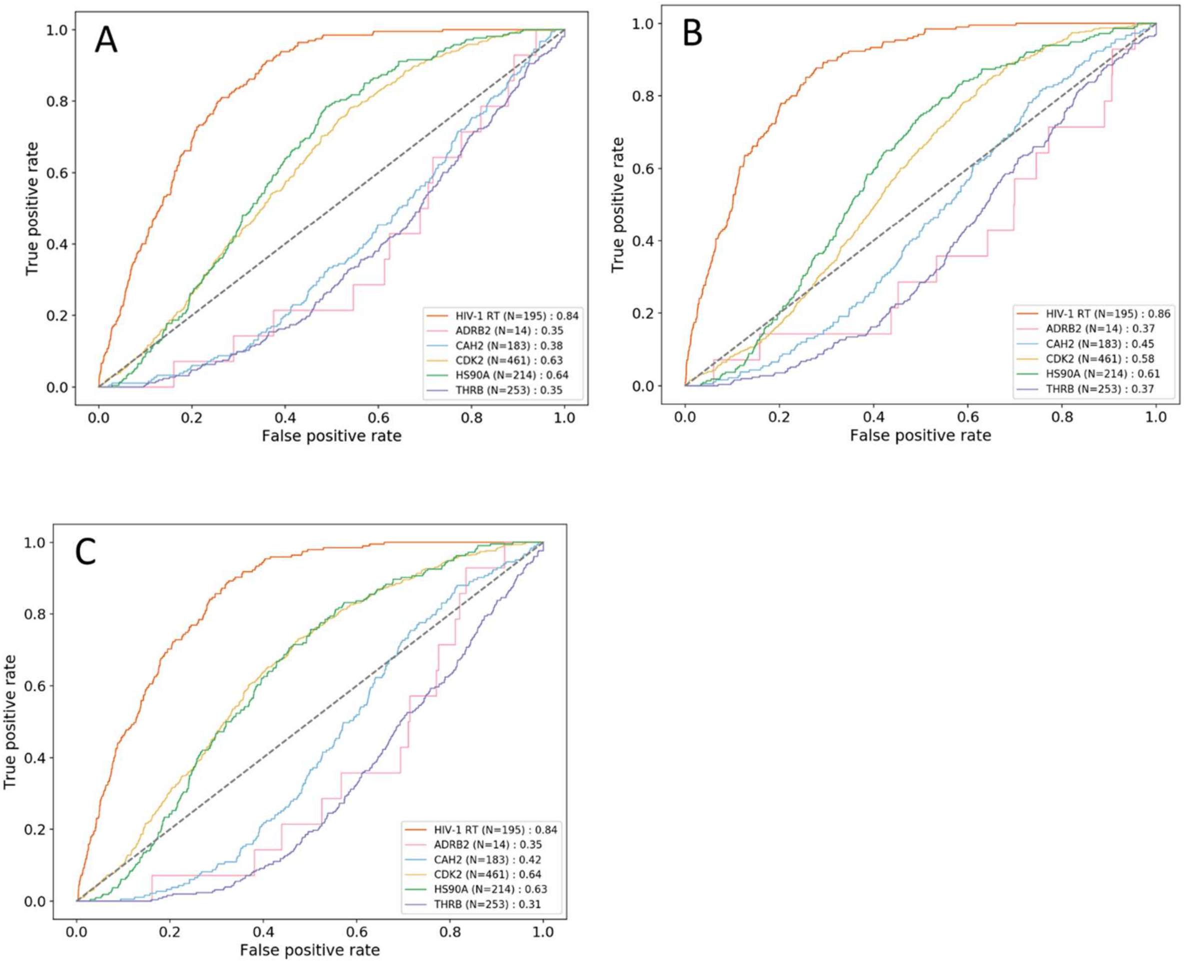


**Figure S1.** Receiver operating characteristic (ROC) curves derived from ProCare similarity scores between sc-PDB subpockets and three TNF-α cavities (6OOY, 6OOZ, 6OP0). For each target (HIV-1 RT, HIV-1 reverse transcriptase; ADRB2, β2 adrenergic receptor; CAH2, carbonic anhydrase; CDK2, cyclin-dependent kinase 2; HSP90A, heat shock protein 90α; THRB, thrombin), the hypothesis is made that its cavity is similar to that of TNF-α and the area under the ROC curve of the corresponding classification is computed. The diagonal black dashed line corresponds to the performance of a random classifier (ROCAUC = 0.50). Number of subpockets for each target is given in brackets. **(A)** 6OOY query, **(B)** 6OOZ query and **(C)** 6OP0 query.

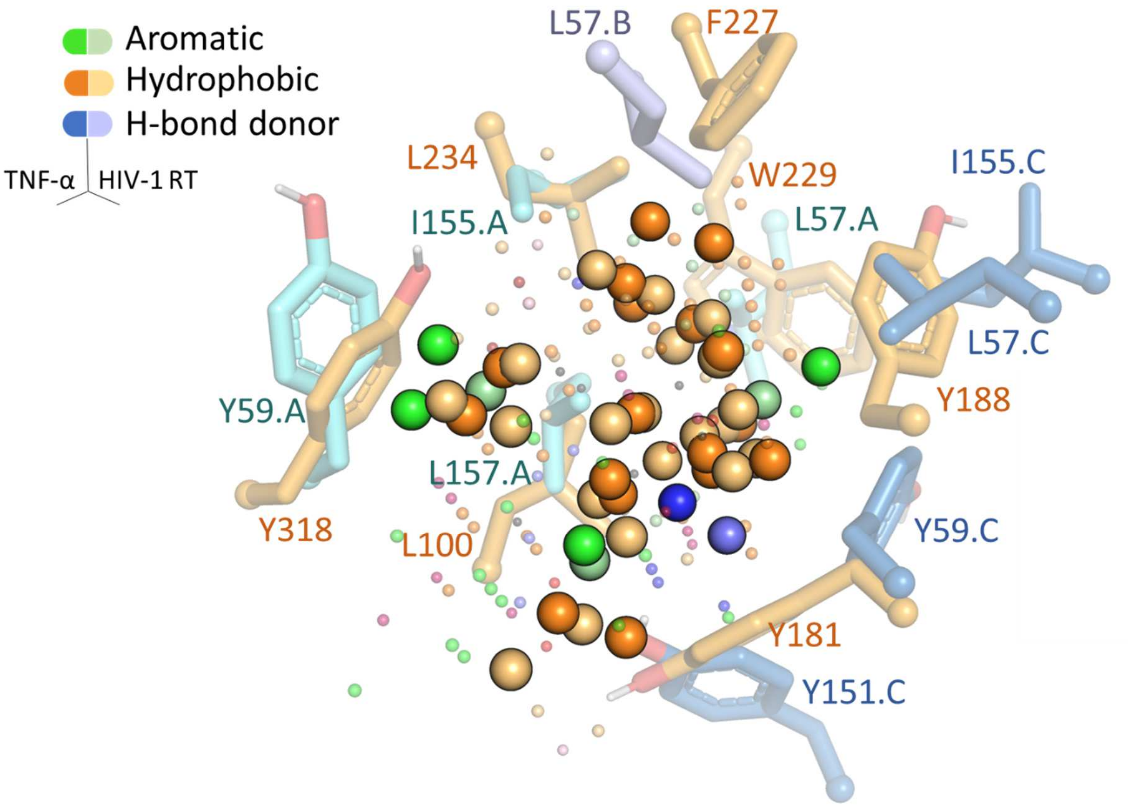


**Figure S2.** ProCare alignment of efavirenz main fragment subpocket (PDB code: 1FKO, HET code: EFZ) onto TNF-α trimer pocket (PDB code: 6OOZ, HET code: A6Y). Matched pharmacophoric points are depicted with dark-colored (TNF-α) and light-colored (HIV-1 RT) large spheres. Small spheres represent pharmacophoric points not considered by the best ProCare alignment.

A

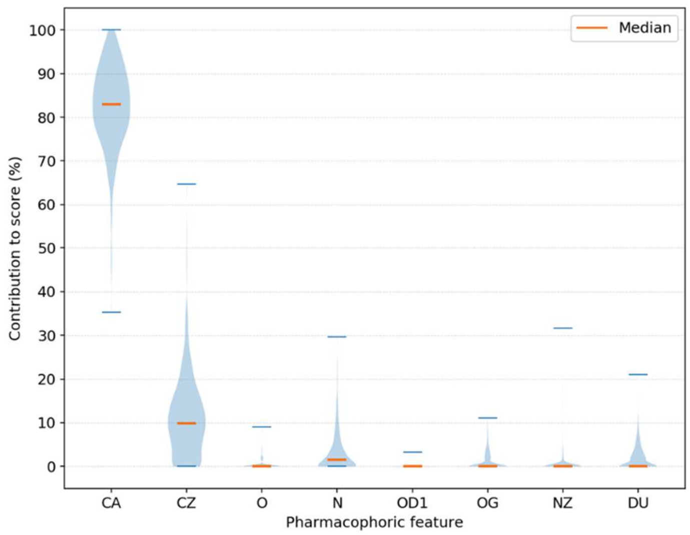


B

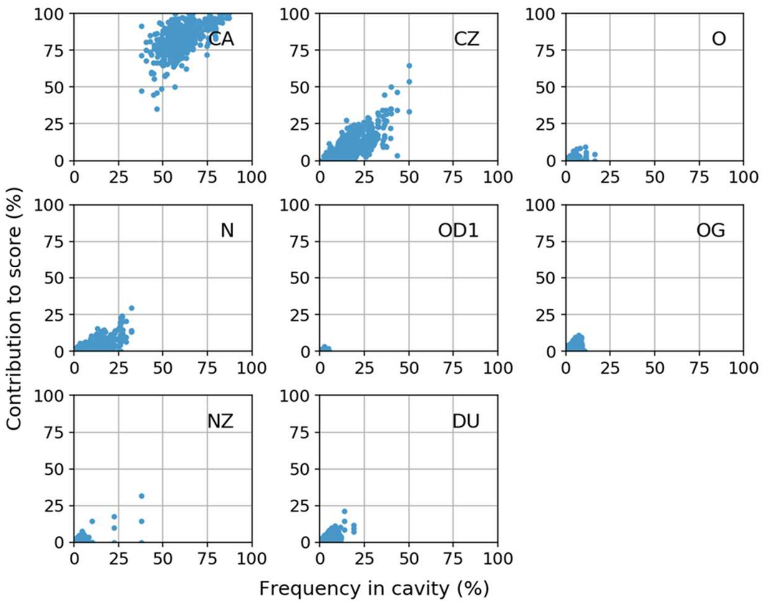


**Figure S3.** Contributions of the eight pharmacophoric features to the ProCare similarity score between HIV-1 RT (PDB ID 1FKO) and TNF-α (PDB ID 6OOZ). CA: hydrophobic, CZ: aromatic, O: h-bond acceptor, N: h-bond donor, OD1: negative, OG: h-bond acceptor and donor, NZ: positive, DU: dummy. **(A)** Aromatic pharmacophoric features are contributing more to the similarity between TNF-α trimer pockets (N=3) and HIV-1 RT subpockets (N = 195) although they are less frequent in the HIV-1 RT subpockets than hydrophobic points **(B).**

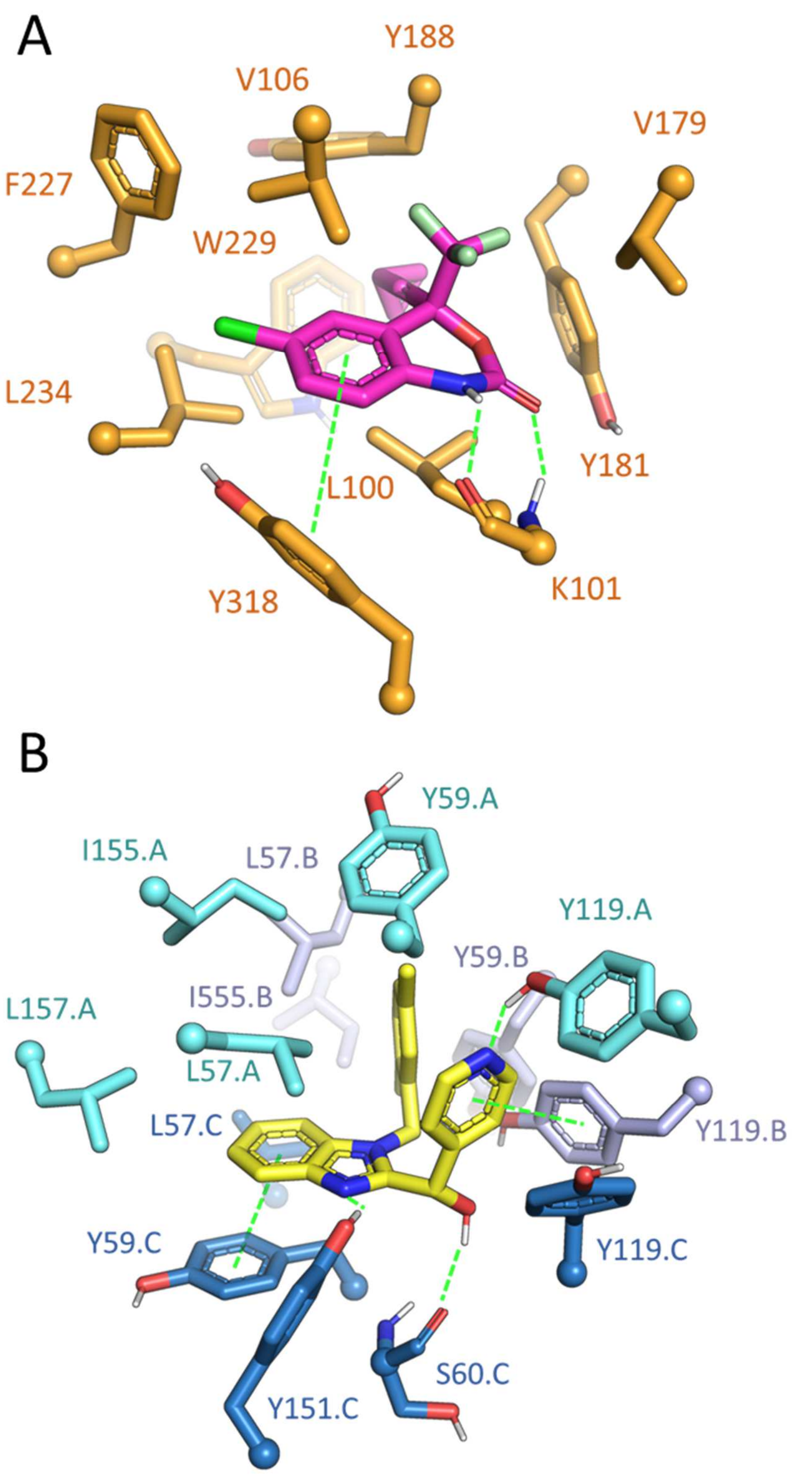


**Figure S4.** Non-covalent interactions between **(A)** efavirenz and HIV-1 RT (PDB ID 1FKO, HET code: EFZ) and **(B)** UCB-5307 and TNF-α trimer (PDB ID 6OOZ, HET code: A6Y).

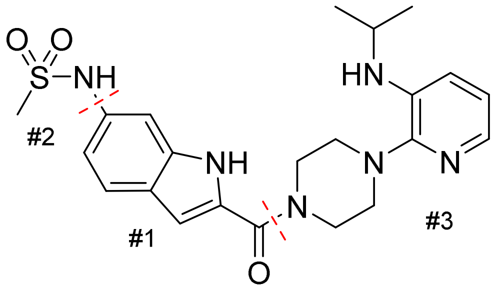


**Figure S5.** Manual fragmentation of delavirdine (PDB code: 1KLM, HET code: SPP) in three fragments (#1 to #3).

**Table S1.** sc-PDB subpockets sorted by decreased ProCare similarity to the inner cavity of human TNF-α (PDB code: 6OOY)

| Cavity ID[a] | Protein name (Uniprot) | Score[b] | Rank |
|---|---|---|---|
| 4f9y_GG5_1_3 | mitogen-activated protein kinase 14 | 0.7679 | 1 |
| 1mz9_VDY_2_1 | cartilage oligomeric matrix protein | 0.7673 | 2 |
| 1oz1_FPH_1_2 | mitogen-activated protein kinase 14 | 0.7634 | 3 |
| 3g9n_J88_1_2 | mitogen-activated protein kinase 10 | 0.7527 | 4 |
| 4fyn_0VE_1_2 | tyrosine-protein kinase syk | 0.7504 | 5 |
| 4kb8_1QN_3_1 | casein kinase i isoform delta | 0.7358 | 6 |
| 3k3j_I46_2_1 | mitogen-activated protein kinase 14 | 0.7338 | 7 |
| 2xj1_XJ1_1_2 | serine/threonine-protein kinase pim-1 | 0.7303 | 8 |
| 4tuv_CPZ_1_1 | cytochrome p450 119 | 0.7303 | 9 |
| 1mr9_ACO_3_2 | streptogramin a acetyltransferase | 0.7301 | 10 |
| 2fze_APR_1_1 | alcohol dehydrogenase class-3 | 0.728 | 11 |
| 4iwc_1GV_2_1 | estrogen receptor | 0.726 | 12 |
| 1ncr_W11_1_2 | human rhinovirus 16 | 0.7256 | 13 |
| 2ykm_YKN_1_2 | HIV-1 reverse transcriptase | 0.7242 | 14 |
| 4a7c_E46_1_1 | serine/threonine-protein kinase pim-1 | 0.7234 | 15 |
| 4wm7_W11_1_2 | capsid protein vp0 | 0.7216 | 16 |
| 3q2a_PAB_2_1 | toluene-4-monooxygenase system, hydroxylase component subunit alpha | 0.7209 | 17 |
| 3bqr_4RB_1_1 | death-associated protein kinase 3 | 0.7193 | 18 |
| 4ccb_OFG_1_4 | alk tyrosine kinase receptor | 0.7191 | 19 |
| 3fc1_52P_1_1 | mitogen-activated protein kinase 14 | 0.7137 | 20 |
| 2uzt_SS3_1_2 | camp-dependent protein kinase catalytic subunit alpha | 0.7126 | 21 |
| 4ewq_MWL_2_3 | mitogen-activated protein kinase 14 | 0.7122 | 22 |
| 4zhx_C1V_1_2 | 5'-amp-activated protein kinase catalytic subunit alpha-2 | 0.7122 | 23 |
| 4ogi_R78_2_2 | bromodomain-containing protein 4 | 0.7115 | 24 |
| 3roc_29A_1_2 | mitogen-activated protein kinase 14 | 0.7093 | 25 |
| 2l1r_SXK_1_2 | troponin c, slow skeletal and cardiac muscles | 0.7071 | 26 |
| 4h98_14Q_2_3 | dihydrofolate reductase | 0.7059 | 27 |
| 1lwc_NVP_1_1 | HIV-1 reverse transcriptase | 0.7024 | 28 |
| 3iw7_IPK_1_1 | mitogen-activated protein kinase 14 | 0.7024 | 29 |
| 2prh_238_2_2 | dihydroorotate dehydrogenase (quinone), mitochondrial | 0.7023 | 30 |
| 3hll_I45_1_1 | mitogen-activated protein kinase 14 | 0.7023 | 31 |
| 2vg7_NNI_1_1 | HIV-1 reverse transcriptase | 0.7022 | 32 |
| 1lwc_NVP_1_2 | HIV-1 reverse transcriptase | 0.6985 | 33 |
| 1ouk_084_1_3 | mitogen-activated protein kinase 14 | 0.6981 | 34 |
| 2xiy_XIY_1_1 | serine/threonine-protein kinase pim-1 | 0.6981 | 35 |

| | | | |
|---|---|---|---|
| 4k33_ACP_1_2 | fibroblast growth factor receptor 3 | 0.698 | 36 |
| 3umw_596_1_2 | serine/threonine-protein kinase pim-1 | 0.6978 | 37 |
| 4nkw_PLO_4_1 | steroid 17-alpha-hydroxylase/17,20 lyase | 0.6975 | 38 |
| 2iok_IOK_1_3 | estrogen receptor | 0.6969 | 39 |
| 2qd9_LGF_1_2 | mitogen-activated protein kinase 14 | 0.6967 | 40 |
| 2hnd_NVP_1_1 | HIV-1 reverse transcriptase | 0.6964 | 41 |
| 5av4_GEN_1_2 | death-associated protein kinase 1 | 0.6964 | 42 |
| 4zth_GG5_1_2 | mitogen-activated protein kinase 14 | 0.6961 | 43 |
| 3vs2_VSB_2_3 | tyrosine-protein kinase hck | 0.6951 | 44 |
| 4r3c_GG5_1_3 | mitogen-activated protein kinase 14 | 0.6951 | 45 |
| 4q5h_ANP_1_2 | protein kinase ospg | 0.6939 | 46 |
| 5awm_ANP_1_2 | stress-activated protein kinase jnk | 0.6936 | 47 |
| 4anq_VGH_1_2 | alk tyrosine kinase receptor | 0.6933 | 48 |
| 1mp0_NAD_2_2 | alcohol dehydrogenase class-3 | 0.6922 | 49 |
| 4anv_751_1_1 | phosphatidylinositol 4,5-bisphosphate 3-kinase catalytic subunit gamma isoform | 0.6918 | 50 |
| 2bxo_OPB_1_2 | albumin | 0.6914 | 51 |
| 1nav_IH5_1_1 | thyroid hormone receptor alpha | 0.6905 | 52 |
| 3fyw_XCF_1_3 | dihydrofolate reductase | 0.6903 | 53 |
| 3lp0_NVP_2_1 | HIV-1 reverse transcriptase | 0.6897 | 54 |
| 1adc_PAD_1_2 | alcohol dehydrogenase e chain | 0.6892 | 55 |
| 2yis_I46_2_1 | mitogen-activated protein kinase 14 | 0.689 | 56 |
| 4uun_NAI_2_2 | l-lactate dehydrogenase | 0.6882 | 57 |
| 4fl2_ANP_1_2 | tyrosine-protein kinase syk | 0.6876 | 58 |
| 4kb8_1QN_3_2 | casein kinase i isoform delta | 0.6874 | 59 |
| 1pjc_NAD_1_2 | alanine dehydrogenase | 0.687 | 60 |
| 3hl7_I47_1_2 | mitogen-activated protein kinase 14 | 0.687 | 61 |
| 4l0q_NAD_1_2 | alcohol dehydrogenase class-3 | 0.6864 | 62 |
| 4loo_SB4_1_2 | mitogen-activated protein kinase 14 | 0.6864 | 63 |
| 3go6_ADP_1_2 | ribokinase | 0.6833 | 64 |
| 3wze_BAX_1_1 | vascular endothelial growth factor receptor 2 | 0.6833 | 65 |
| 2xiz_XIZ_1_1 | serine/threonine-protein kinase pim-1 | 0.6822 | 66 |
| 3rsr_N5P_1_1 | ribonucleoside-diphosphate reductase large chain 1 | 0.6816 | 67 |
| 1jkl_ANP_1_2 | death-associated protein kinase 1 | 0.6807 | 68 |
| 3bxz_ADP_2_2 | protein translocase subunit seca | 0.6807 | 69 |
| 3uyt_0CK_3_3 | casein kinase i isoform delta | 0.6807 | 70 |
| 1qiw_DPD_2_2 | calmodulin | 0.6806 | 71 |
| 4mbl_26L_1_2 | serine/threonine-protein kinase pim-1 | 0.6802 | 72 |
| 4kbc_1QJ_1_1 | casein kinase i isoform delta | 0.6797 | 73 |
| 1vru_AAP_1_2 | HIV-1 reverse transcriptase | 0.6791 | 74 |
| 1f0y_NAD_2_2 | hydroxyacyl-coenzyme a dehydrogenase, mitochondrial | 0.6786 | 75 |

| | | | |
|---|---|---|---|
| 4hds_IPH_1_1 | n(1)-alpha-phosphoribosyltransferase | 0.6786 | 76 |
| 4mzu_COA_22_2 | wxcm-like protein | 0.6786 | 77 |
| 3gc7_B45_1_1 | mitogen-activated protein kinase 14 | 0.6782 | 78 |
| 3qf9_NM8_1_2 | serine/threonine-protein kinase pim-1 | 0.678 | 79 |
| 3rr3_FLR_3_2 | prostaglandin g/h synthase 2 | 0.678 | 80 |
| 4ix6_ADP_1_2 | protein kinase domain-containing protein | 0.6771 | 81 |
| 1pf9_ADP_2_2 | 60 kda chaperonin | 0.6761 | 82 |
| 2bu7_TF3_1_2 | [pyruvate dehydrogenase (acetyl-transferring)] kinase isozyme 2, mitochondrial | 0.6761 | 83 |
| 5ani_ES4_1_1 | cyclin-dependent kinase 2 | 0.6761 | 84 |
| 1tuv_VK3_1_1 | probable quinol monooxygenase ygin | 0.6754 | 85 |
| 2zm1_KSF_1_2 | tyrosine-protein kinase lck | 0.6752 | 86 |
| 3bea_IXH_1_3 | angiopoietin-1 receptor | 0.6752 | 87 |
| 4hur_ACO_3_1 | virginiamycin a acetyltransferase | 0.6752 | 88 |
| 5dr2_ATP_1_2 | aurora kinase a | 0.6752 | 89 |
| 5dgz_L20_1_1 | serine/threonine-protein kinase pim-1 | 0.674 | 90 |
| 4iu7_1GM_1_1 | estrogen receptor | 0.6736 | 91 |
| 2pnu_ENM_1_1 | androgen receptor | 0.6736 | 92 |
| 3hvc_GG5_1_3 | mitogen-activated protein kinase 14 | 0.6736 | 93 |
| 3wwm_ADP_1_2 | [lysw]-aminoadipate kinase | 0.6736 | 94 |
| 3znr_NU9_1_3 | histone deacetylase 7 | 0.6736 | 95 |
| 3q7d_NPX_1_1 | prostaglandin g/h synthase 2 | 0.6726 | 96 |
| 3fkn_FKN_1_1 | mitogen-activated protein kinase 14 | 0.6723 | 97 |
| 4dgm_AGI_1_1 | casein kinase ii subunit alpha | 0.6721 | 98 |
| 4i5h_G17_1_1 | mitogen-activated protein kinase 1 | 0.6721 | 99 |
| 3t9i_3T9_1_1 | serine/threonine-protein kinase pim-1 | 0.6715 | 100 |
| ... | ... | ... | ... |
| 5je3_SAH_2_2 | class I sam-dependent methyltransferase | 0.0000 | 31570 |

[a] Cavity ID (PDB_HET_C_M) is inferred from the cognate target PDB identifier (PDB), the corresponding ligand chemical component (HET), the target cavity identifier (C), and the fragment number (N).

[b] ProCare similarity score. A value above 0.47 corresponds to statistically significant similarity (p-value < 0.05) between the pair of pockets under investigation [17].

**Table S2.** PDB entries describing non-nucleoside inhibitors bound to HIV-1 reverse transcriptase

| PDB ID[a] | sc-PDB Site[b] | HET[c] | Uniprot AC[d] |
|---|---|---|---|
| 1C0T | 1 | BM1 | P04585 |
| 1C0U | 1 | BM5 | P04585 |
| 1C1B | 1 | GCA | P04585 |
| 1C1C | 1 | 612 | P04585 |
| 1EP4 | 1 | S11 | P04585 |
| 1FK9 | 1 | EFZ | P04585 |
| 1FKO | 1 | EFZ | P04585 |
| 1FKP | 1 | NVP | P04585 |
| 1JKH | 1 | EFZ | P04585 |
| 1JLA | 1 | TNK | P04585 |
| 1JLB | 1 | NVP | P04585 |
| 1JLC | 1 | FTC | P04585 |
| 1JLF | 1 | NVP | P04585 |
| 1JLG | 1 | UC1 | P04585 |
| 1JLQ | 1 | SBN | P04585 |
| 1KLM | 1 | SPP | P04585 |
| 1LW0 | 1 | NVP | P04585 |
| 1LW2 | 1 | U05 | P04585 |
| 1LWC | 1 | NVP | P04585 |
| 1LWE | 1 | NVP | P04585 |
| 1LWF | 1 | NVP | P04585 |
| 1REV | 1 | TB9 | P04585 |
| 1RT1 | 1 | MKC | P04585 |
| 1RT2 | 1 | TNK | P04585 |
| 1RT3 | 1 | U05 | P04585 |
| 1RT4 | 1 | UC1 | P04585 |
| 1RT5 | 1 | UC2 | P04585 |
| 1RT6 | 1 | UC3 | P04585 |
| 1RT7 | 1 | UC4 | P04585 |
| 1RTH | 1 | U05 | P04585 |
| 1RTI | 1 | HEF | P04585 |
| 1S1T | 1 | UC1 | P04585 |
| 1S1U | 1 | NVP | P04585 |
| 1S1V | 1 | TNK | P04585 |
| 1S1W | 1 | UC1 | P04585 |
| 1S1X | 1 | NVP | P04585 |
| 1TKT | 1 | H12 | P04585 |
| 1TKX | 1 | GWB | P04585 |
| 1TKZ | 1 | H16 | P04585 |
| 1TL1 | 1 | H18 | P04585 |
| 1TL3 | 1 | H20 | P04585 |

| | | | |
|---|---|---|---|
| 1VRT | 1 | NVP | P04585 |
| 1VRU | 1 | AAP | P04585 |
| 2HND | 1 | NVP | P04585 |
| 2HNY | 1 | NVP | P04585 |
| 2HNZ | 1 | PC0 | P04585 |
| 2OPP | 1 | HBQ | P04585 |
| 2OPQ | 1 | HBQ | P04585 |
| 2OPR | 1 | HBQ | P04585 |
| 2OPS | 1 | HBQ | P04585 |
| 2RF2 | 1 | MRX | P04585 |
| 2RKI | 1 | TT1 | P04585 |
| 2WON | 1 | ZZE | P04585 |
| 2YNF | 1 | WHU | P04585 |
| 2YNH | 1 | EUR | P04585 |
| 3C6T | 1 | M14 | P04585 |
| 3C6U | 1 | M22 | P04585 |
| 3DI6 | 1 | PDZ | P04585 |
| 3DLE | 1 | GFA | P04585 |
| 3DLG | 1 | GWE | P04585 |
| 3DOK | 1 | GWJ | A7YKL0 |
| 3DRP | 1 | R8E | P04585 |
| 3DRR | 1 | R8E | P04585 |
| 3DYA | 1 | PZL | P04585 |
| 3E01 | 1 | PZ2 | P04585 |
| 3I0R | 1 | RT3 | P04585 |
| 3I0S | 1 | RT7 | P04585 |
| 3LAK | 1 | KR1 | P04585 |
| 3LAL | 1 | KRV | P04585 |
| 3LAM | 1 | KRP | P04585 |
| 3LAN | 1 | KBT | P04585 |
| 3LP0 | 2 | NVP | P04585 |
| 3LP1 | 2 | NVP | P04585 |
| 3LP2 | 1 | LP9 | P04585 |
| 3M8P | 1 | 65B | P04585 |
| 3M8Q | 1 | DJZ | P04585 |
| 3MEC | 1 | 65B | P04585 |
| 3MED | 1 | 65B | P04585 |
| 3MEE | 1 | T27 | P04585 |
| 3MEG | 1 | T27 | P04585 |
| 3NBP | 1 | JGZ | P04585 |
| 3QIP | 1 | NVP | P04585 |
| 3T19 | 1 | 5MA | P04585 |
| 3T1A | 1 | 5MA | P04585 |

| | | | |
|---|---|---|---|
| 3TAM | 1 | M06 | P04585 |
| 4I7F | 1 | NVE | P04585 |
| 2BE2 | 1 | R22 | P03366 |
| 4H4O | 1 | 506 | P03366 |
| 3DMJ | 1 | GWE | A7YKL0 |
| 2B5J | 1 | 3AC | P03366 |
| 4ID5 | 1 | T27 | P03366 |
| 3HVT | 1 | NVP | P03366 |
| 2VG5 | 1 | NNC | P03366 |
| 1TVR | 1 | TB9 | P03366 |
| 3QLH | 2 | T27 | P03366 |
| 2BAN | 1 | 357 | P03366 |
| 4G1Q | 1 | T27 | P03366 |
| 4I2Q | 1 | 1BT | P03366 |
| 4RW4 | 1 | 494 | P03366 |
| 2JLE | 1 | I15 | Q72547 |
| 5CYQ | 1 | T27 | P03366 |
| 1S9E | 1 | ADB | P03366 |
| 3IS9 | 1 | AC7 | P03366 |
| 4RW6 | 1 | 494 | P03366 |
| 2ZD1 | 1 | T27 | P03366 |
| 1S6Q | 1 | TPB | P03366 |
| 2ZE2 | 1 | T27 | P03366 |
| 4MFB | 1 | 29T | P03366 |
| 3IRX | 1 | UDR | P03366 |
| 4H4M | 1 | 494 | P03366 |
| 4KKO | 1 | 1RE | P03366 |
| 4IFV | 1 | T27 | P03366 |
| 3BGR | 1 | T27 | P03366 |
| 2YKM | 1 | YKN | P03366 |
| 3DOL | 1 | GWI | A7YKL0 |
| 4KFB | 2 | T27 | P03366 |
| 4LSL | 1 | 1YQ | P03366 |
| 4I2P | 1 | G73 | P03366 |
| 4KO0 | 1 | JLJ | P03366 |
| 2IC3 | 1 | HBY | P03366 |
| 2VG7 | 1 | NNI | P03366 |
| 4IG3 | 1 | T27 | P03366 |

[a] PDB identifier (https://www.rcsb.org/)

[b] sc-PDDB pocket number (http://bioinfo-pharma.u-strasbg.fr/scPDB/)

[c] PDB chemical component identifier (https://www.ebi.ac.uk/pdbe-srv/pdbechem/)

[d] UniProt accession number (https://www.uniprot.org/)

**Table S3.** Comparison of delavirdine subpockets, resulting from manual fragmentation, with TNF-α trimer pockets.

| PDB/HET code | Fragment # | TNF-α PDB entry | ProCare score | Rank[a] |
|---|---|---|---|---|
| 1KLM/SPP | 1 | 6OOY | 0.328 | 588 |
| 1KLM/SPP | 2 | 6OOY | 0.599 | 113 |
| 1KLM/SPP | 3 | 6OOY | 0.283 | 593 |
| 1KLM/SPP | 1 | 6OOZ | 0.361 | 581 |
| 1KLM/SPP | 2 | 6OOZ | 0.570 | 174 |
| 1KLM/SPP | 3 | 6OOZ | 0.416 | 549 |
| 1KLM/SPP | 1 | 6OP0 | 0.342 | 586 |
| 1KLM/SPP | 2 | 6OP0 | 0.534 | 272 |
| 1KLM/SPP | 3 | 6OP0 | 0.130 | 594 |

[a] Rank after adding delavirdine fragment scores to the ProCare screening results that yielded a total of 594 pairwise scores.

**Table S4.** Dissociation constant ($K_D$) of three HIV-1 RT inhibitor binding to human soluble TNF-α, according to MST experimental conditions.

| HIV-1 RT Inhibitor | TNF concentration nM | DMSO concentration (%) in MST buffer | Tween-20 concentration in MST buffer | Incubation time min | MST power % | $K_D$ ± CI[a] µM |
|---|---|---|---|---|---|---|
| efavirenz | 220 | 5.0 | 0.05 | 5 | 40 | 45 ± 9 |
| efavirenz | 220 | 5.0 | 0.05 | 5 | 80 | 47 ± 12 |
| efavirenz | 220 | 5.0 | 0.01 | 5 | 80 | 26 ± 5 |
| efavirenz | 220 | 5.0 | 0.01 | 30 | 80 | 27 ± 6 |
| efavirenz | 220 | 2.5 | 0.01 | 30 | 80 | 11 ± 3 |
| efavirenz | 170 | 1.3 | 0.01 | 20 | 40 | 17 ± 5 |
| efavirenz | 170 | 1.3 | 0.01 | 20 | 80 | 24 ± 4 |
| efavirenz | 340 | 1.3 | 0.01 | 15 | 40 | 24 ± 8[b] |
| efavirenz | 340 | 1.3 | 0.01 | 15 | 80 | 38 ± 5 |
| | | | | | | |
| delavirdine | 220 | 5.0 | 0.05 | 5 | 40 | 203 ± 143 |
| delavirdine | 220 | 5.0 | 0.01 | 5 | 40 | 84 ± 57 |
| delavirdine | 170 | 1.3 | 0.01 | 5 | 40 | 90 ± 50 |
| delavirdine | 170 | 1.3 | 0.01 | 60 | 20 | 81 ± 31 |
| delavirdine | 170 | 1.3 | 0.01 | 15 | 20 | 69 ± 23 |
| delavirdine | 340 | 1.3 | 0.01 | 15 | 20 | 39 ± 9[b] |
| delavirdine | 340 | 1.3 | 0.01 | 120 | 20 | 56 ± 20 |
| | | | | | | |
| nevirapine | 220 | 5.0 | 0.05 | 5 | 40 | no signal |
| nevirapine | 340 | 1.3 | 0.01 | 20 | 40 | no signal |

[a] CI: 68.3% confidence interval

[b] MST measure with the highest signal to noise ratio

**Table S5.** CHEMBL entries describing HIV-1 RT non-nucleoside inhibitors.

Available at https://github.com/kimeguida/ProCare_TNF

**Table S6.** Customized rules for OpenEye Filter ionization.

```
MIN_MOLWT     1        "Minimum molecular weight"
MAX_MOLWT     15000        "Maximum molecular weight"
MIN_NUM_HVY   0        "Minimum number of heavy atoms"
MAX_NUM_HVY   2500        "Maximum number of heavy atoms"
MIN_RING_SYS    0     "Minumum number of ring systems"
MAX_RING_SYS    50     "Maximum number of ring systems"
MIN_RING_SIZE    0     "Minimum atoms in any ring system"
MAX_RING_SIZE    200     "Maximum atoms in any ring system"
MIN_CON_NON_RING    0     "Minimum number of connected non-ring atoms"
MAX_CON_NON_RING    190     "Maximum number of connected non-ring atoms"
MIN_FCNGRP      0     "Minimum number of functional groups"
MAX_FCNGRP      70     "Maximum number of functional groups"
MIN_UNBRANCHED   0     "Minimum number of connected unbranched non-ring atoms"
MAX_UNBRANCHED   130     "Maximum number of connected unbranched non-ring atoms"
MIN_CARBONS     0     "Minimum number of carbons"
MAX_CARBONS     410     "Maximum number of carbons"
MIN_HETEROATOMS    0     "Minimum number of heteroatoms"
MAX_HETEROATOMS    140     "Maximum number of heteroatoms"
MIN_Het_C_Ratio    0.04     "Minimum heteroatom to carbon ratio"
MAX_Het_C_Ratio    40.0     "Maximum heteroatom to carbon ratio"
MIN_HALIDE_FRACTION     0.0     "Minimum Halide Fraction"
MAX_HALIDE_FRACTION     0.99     "Maximum Halide Fraction"
#count ring degrees of freedom = (#BondsInRing) - 4 - (RigidBondsInRing) - (BondsSharedWithOtherRings)
#must be >= 0, from JCAMD 14:251-265,2000.
ADJUST_ROT_FOR_RING     true     "BOOLEAN for whether to estimate degrees of freedom in rings"
MIN_ROT_BONDS    0     "Minimum number of rotatable bonds"
MAX_ROT_BONDS    160     "Maximum number of rotatable bonds"
MIN_RIGID_BONDS    0     "Minimum number of rigid bonds"
MAX_RIGID_BONDS    550     "Maximum number of rigid bonds"
MIN_HBOND_DONORS  0     "Minimum number of hydrogen-bond donors"
MAX_HBOND_DONORS  90     "Maximum number of hydrogen-bond donors"
MIN_HBOND_ACCEPTORS  0     "Minimum number of hydrogen-bond acceptors"
MAX_HBOND_ACCEPTORS  130     "Maximum number of hydrogen-bond acceptors"
MIN_LIPINSKI_DONORS  0     "Minimum number of hydrogens on O & N atoms"
MAX_LIPINSKI_DONORS  60     "Maximum number of hydrogens on O & N atoms"
MIN_LIPINSKI_ACCEPTORS  0     "Minimum number of oxygen & nitrogen atoms"
MAX_LIPINSKI_ACCEPTORS  140     "Maximum number of oxygen & nitrogen atoms"
MIN_COUNT_FORMAL_CRG    0     "Minimum number formal charges"
MAX_COUNT_FORMAL_CRG    40     "Maximum number of formal charges"
MIN_SUM_FORMAL_CRG   -20     "Minimum sum of formal charges"
MAX_SUM_FORMAL_CRG    20     "Maximum sum of formal charges"
MIN_CHIRAL_CENTERS   0   "Minimum chiral centers"
MAX_CHIRAL_CENTERS   100  "Maximum chiral centers"
MIN_XLOGP     -30.0     "Minimum XLogP"
MAX_XLOGP      60.85     "Maximum XLogP
```

```
#choices are insoluble<poorly<moderately<soluble<very<highly
MIN_SOLUBILITY     insoluble "Minimum solubility"
PSA_USE_SandP   false    "Count S and P as polar atoms"
MIN_2D_PSA     0.0     "Minimum 2-Dimensional (SMILES) Polar Surface Area"
MAX_2D_PSA     2050.0    "Maximum 2-Dimensional (SMILES) Polar Surface Area"
AGGREGATORS    false     "Eliminate known aggregators"
PRED_AGG      false     "Eliminate predicted aggregators"
#secondary filters (based on multiple primary filters)
GSK_VEBER     false     "PSA>140 or >10 rot bonds"
MAX_LIPINSKI   5       "Maximum number of Lipinski violations"
MIN_ABS       0.01     "Minimum probability F>10% in rats"
PHARMACOPIA    false     "LogP > 5.88 or PSA > 131.6"
ALLOWED_ELEMENTS  H,C,N,O,F,P,S,Cl,Br,I,B
ELIMINATE_METALS Sc,Ti,V,Cr,Mn,Fe,Co,Ni,Cu,Zn,Y,Zr,Nb,Mo,Tc,Ru,Rh,Pd,Ag,Cd
```
